# CGRP Enhances the Regeneration of Bone Defects by Regulating Bone Marrow Mesenchymal Stem Cells Through Promoting ANGPTL4 Secretion by Bone Blood Vessels

**DOI:** 10.1002/advs.202522295

**Published:** 2026-01-05

**Authors:** Qiong Lu, Qiyue Zheng, Zhaokai Zhou, Yajun Chen, Yun Chen, Wenjie Chen, Jiaojiao Wang, Ren Guo, Ren Wu, Lina Chen

**Affiliations:** ^1^ Department of Pharmacology School of Basic Medical Sciences Xi'an Jiaotong University Xi'an Shaanxi China; ^2^ Department of Pharmacy the Second Xiangya Hospital Central South University Changsha Hunan China; ^3^ Institute of Clinical Pharmacy Central South University Changsha Hunan China; ^4^ Department of Urology The Second Xiangya Hospital of Central South University Changsha Hunan China; ^5^ College of Traditional Chinese Medicine Guangzhou University of Chinese Medicine Guangzhou Guangdong China; ^6^ Department of Pharmacy The Third Xiangya Hospital Central South University Changsha Hunan China; ^7^ Department of Orthopedics the Second Xiangya Hospital of Central South University Changsha Hunan China

**Keywords:** ANGPTL4, bone defect, bone marrow mesenchymal stem cells, CGRP, human microvascular endothelial cells, single‐cell RNA sequencing, vascular–osteogenic coupling

## Abstract

Bone angiogenesis is important for bone formation and regeneration after bone injury. Endothelial‐derived angiogenic factors are key signal transducers in the bone microenvironment and maintain vascular–osteogenic coupling during bone regeneration. CGRP, a bone sensory neuron‐derived peptide, contributes to bone formation, but the potential mechanism by which it improves bone regeneration via angiogenesis is unclear. Here, we demonstrate that CGRP may contribute to bone repair in the elderly, as human CGRP levels are inversely proportional to age and proportional to bone mass in clinical data and bulk transcriptome data. Based on single‐cell RNA sequencing data and experimental analyses, CGRP is found to promote the angiogenesis of human microvascular endothelial cell line‐1 in vitro through the FAK–AKT–VEGF pathway. CGRP gene deletion in mice reduced bone vascular density and bone mass, and delayed angiogenesis and bone regeneration at the bone defect site. Recombinant CGRP restored bone repair after defect introduction. It also promoted Angptl4 secretion by bone vascular endothelial cells, thereby driving osteogenic differentiation of bone marrow mesenchymal stem cells and enhancing bone regeneration after bone injury. Treatment with recombinant Angptl4 enhanced bone healing in a mouse bone defect model. These integrated analysis reveal the important role and mechanism of CGRP in vascular‐mediated osteogenesis, suggesting a novel therapeutic strategy for promoting bone regeneration.

## Graphical abstract

CGRP interacts with HMEC‐1 by binding to surface receptors, influencing the downstream FAK/AKT/VEGF signaling pathway, and promoting the secretion of ANGPTL4 from vascular endothelial cells into the bone microenvironment. This, in turn, regulates the differentiation of BMSCs into osteoblasts, thus promoting bone formation.

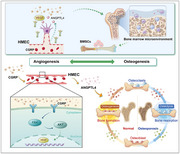

**Abbreviations**
ANGPTL4: angiopoietin‐related protein 4
BMSC: bone marrow mesenchymal stem cell
BMD: bone mineral density
EC: endothelial cell
ELISA: enzyme‐linked immunosorbent assay
FAK: focal adhesion kinase
GEO: Gene Expression Omnibus
HMEC‐1: human microvascular endothelial cell line‐1
GO: Gene Ontology
GSEA: Gene Set Enrichment Analysis
MSC: mesenchymal stem cells
LC‐MS/MS: liquid chromatography‐tandem mass spectrometry
KEGG: Kyoto Encyclopedia of Genes and Genomes
OLC: osteolineage cell
PCR: polymerase chain reaction
micro‐CT: micro‐computed tomography
PBS: phosphate‐buffered saline
LEPR: leptin receptor
RT‐qPCR: Quantitative reverse transcription PCR
RNA‐seq: RNA sequencing
scRNA‐seq: single‐cell RNA sequencing
PCA: principal component analysis
SNN: shared nearest neighbor
VEGFA: vascular endothelial growth factor A
WT: wild‐type; Cgrp‐/‐: Cgrp knockout

## 1 Introduction

Bone, as a dynamic tissue, continuously undergoes remodeling through resorption and formation. Nevertheless, in osteoporotic conditions, an imbalance in bone homeostasis results in the loss of bone mass, thereby increasing the risk of fragility fractures. Osteoporotic fragility fractures increase the risk of mortality and morbidity [1]. Recent studies have indicated that angiogenesis and bone formation are tightly integrated to maintain bone homeostasis. As the “nutrient channels” of the bone, blood vessels can regulate the growth and metabolism of bone cells in a paracrine fashion and facilitate communication with other lineage cells to enhance the coupling of angiogenesis and osteogenesis, which ensures the growth and development of bone and the maintenance of homeostasis [2, 3, 4].

Numerous studies have reported that CGRP levels peak early after fracture, driving bone formation and blood vessel formation, ultimately leading to bone regeneration [5, 6]. These results suggested that CGRP may regulate osteoporosis and promote bone healing by modulating bone angiogenesis. In our previous study, we also identified CGRP as a key regulator in the age‐related shift from the generation of osteoblasts to that of lipoblasts in bone marrow mesenchymal stem cells (BMSCs) [7]. However, the underlying mechanism through which CGRP promotes angiogenesis–osteogenesis coupling remains unclear.

Angiopoietin‐related protein 4 (ANGPTL4) is a member of the angiopoietin‐like protein family [8]. It has been shown that ANGPTL4 is strongly expressed in osteoblasts on the new bone surface of fracture calluses, and exogenous ANGPTL4 increases the expression of *Runx2* and *Alp* mRNA in differentiated osteoblasts, suggesting that ANGPTL4 is closely related to bone formation [9].

In this study, we examined serum samples from elderly patients with osteoporosis, and found that CGRP expression is associated with bone mass. In addition, our study found that compared to wild‐type (WT) controls, CGRP knockout (CGRP^−/−^) mice exhibited reduced bone angiogenesis and impaired bone formation. Bulk transcriptome data, single‐cell RNA sequencing (scRNA‐seq) data, internal RNA sequencing (RNA‐seq) data, and phosphoproteome data were used to investigate the specific mechanism of CGRP‐promoted bone formation and identified ANGPTL4 as a potential factor in vascular–osteogenic coupling. This study further revealed that CGRP promoted the secretion of ANGPTL4 by human microvascular endothelial cell line‐1 (HMEC‐1) cells and decreased the expression of ANGPTL4 in CGRP^−/−^ mice. These findings indicated a correlation between CGRP and ANGPTL4 expression and the ability of CGRP to stimulate ANGPTL4 secretion by vascular endothelial cells. It was also observed that ANGPTL4 supported the differentiation of BMSCs into osteoblasts and inhibited their adipogenic differentiation. In summary, the comprehensive analysis confirmed the molecular mechanisms by which CGRP prevents osteoporosis and promotes bone regeneration, suggesting its potential as a novel therapeutic target.

## 2 Materials and Methods

### 2.1 Patients and Samples

**FIGURE 1:**
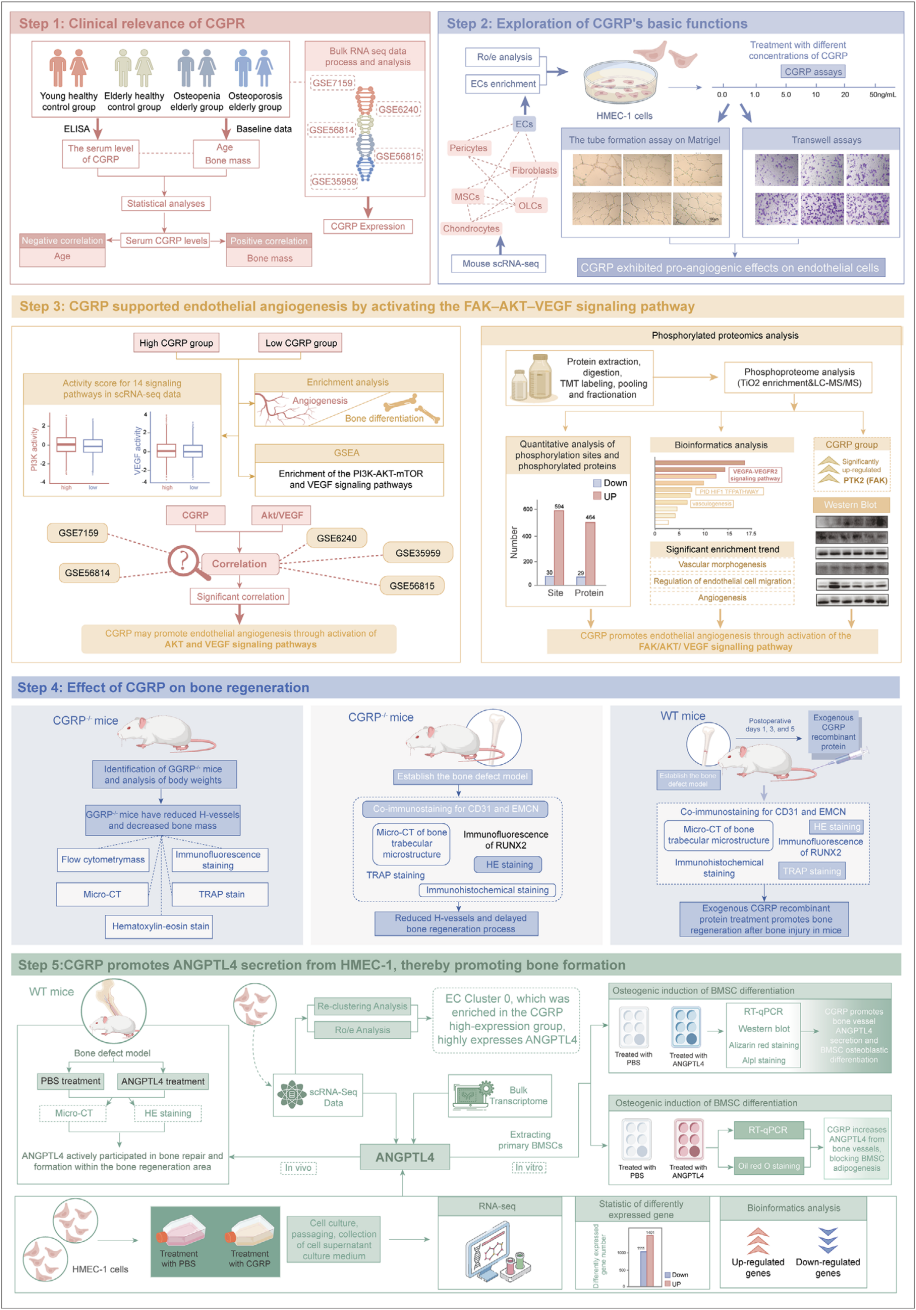
The flowchart of this study.

This study was approved by the Ethics Committee of the Second Xiangya Hospital of Central South University (ethics approval number Kq2208340). Figure 1 illustrates the flowchart of this study. Participants were continuously recruited at the Second Xiangya Hospital of Central South University from August 2023 to February 2024. According to the World Health Organization criteria, osteoporosis was defined as T score ≤−2.5, low bone mass was defined as −2.5 <T score <−1.0, and normal bone mass was defined as T score ≥−1.0. The inclusion criteria for participants included: (1) voluntarily participating in the study and signing informed consent forms, and (2) in accordance with the diagnostic criteria for osteoporosis recommended by the World Health Organization. The exclusion criteria were: (1) patients with malignant tumors or hereditary diseases; (2) endocrine system diseases, such as thyroid and parathyroid gland disorders; (3) impaired function of important organs, such as heart, liver, and kidney; and (4) individuals who were unwilling to participate in the study. Eight individuals each were selected to represent young healthy controls, older healthy controls, older individuals with low bone mass, and older individuals with osteoporosis. The anthropometric characteristics of the participants, such as age and bone mineral density, were recorded. Detailed clinical data from the study participants are shown in Table 1. Bone mineral density was measured by dual‐energy X‐ray absorptiometry. Peripheral blood was collected in the morning on an empty stomach, and left for 1 h at room temperature to coagulate. The serum was collected by centrifugation and stored at −80°C until use.

**TABLE 1:** Baseline characteristics of the eight young subjects.

Number	Age(years)	Gender	Calcium(2.11‐2.52 mmol/L)	Phosphorus(0.85–1.51 mmol/L)
1	37	female	2.24	1.08
2	30	male	2.32	0.98
3	49	male	2.41	1.13
4	35	male	2.45	0.86
5	34	male	2.50	1.11
6	39	male	2.26	0.97
7	36	female	2.39	1.21
8	52	female	2.44	1.16

### 2.2 Bulk RNA‐Seq Data Process and Analysis

Gene expression data and corresponding clinical data from GSE7158 (*n* = 26), GSE35959 (*n* = 19), GSE56814 (*n* = 73), GSE56814 (*n* = 80), GSE56815 (*n* = 80), and GSE62402 (*n* = 10) were downloaded from the Gene Expression Omnibus (GEO, https://www.ncbi.nlm.nih.gov/geo/). GSE7158 dataset conducted a genome‐wide gene differential expression study on circulating monocytes from premenopausal human subjects with extremely low (*n* = 12) and high (*n* = 14) peak bone mass (PBM) [10] GSE35959 dataset represents microarray analysis of multipotent mesenchymal stem cells (MSCs) derived from elderly patients with primary osteoporosis aged 79–94 years (*n* = 5), age‐matched control patients (*n* = 4), and nonosteoporotic middle‐aged (*n* = 5) and elderly patients (*n* = 5) [11] GSE56814 dataset performed microarray analysis of circulating monocytes on 73 Caucasian women (age: 47–56 years), including a group with very high bone mineral density (BMD, *n* = 42) and a low hip BMD group (*n* = 31) [12, 13]. GSE56815 dataset includes circulating monocyte microarray data from 40 subjects with high BMD and 40 subjects with low hip BMD [14] GSE62402 contains circulating monocyte microarray data from 5 subjects with high hip BMD and 5 subjects with low hip BMD [15].

### 2.3 ScRNA‐Seq Data Collection and Processing

In this study, the GSE128423 datasets from non‐hematopoietic bone marrow cells in mouse (*n* = 6) by scRNA‐seq were collected and analyzed [16]. The R package Seurat (version 4.4.0) was used for single‐cell data preprocessing. The top 2000 highly variable genes were identified using the FindVariableFeatures function. The unwanted sources of variation were regressed out using the ScaleData function. Dimensionality reduction was performed using principal component analysis with the first 50 components. The FindNeighbors function was used to construct a shared nearest neighbor graph based on edge weights between cells. In the annotation, each cluster was annotated based on known markers from established literature [16].

### 2.4 Pseudobulk Analysis and Ro/e Analysis

Pseudobulk analysis is an analytical method that aggregates scRNA‐seq (GSE128423) into samples to simulate traditional batch RNA‐seq data, which is used to visualize the distribution of cell fractions across different groups (CGRP high/low groups and Angptl4 high/low groups). Ro/e analysis primarily assesses whether different groups exhibit significant deviations from random expectations in tissue distribution, further indicating the enrichment or depletion of specific cell clusters within particular tissues. Ro/e > 1 signifies that the number of cells in a given cluster exceeds random expectations within that tissue, representing enrichment. Ro/e<1 indicates that the number of cells in a particular cluster within the tissue is lower than the random expectation.

### 2.5 Pathway Activity Analysis and Transcription Factor Activity Analysis

The PROGENy (Pathway RespOnsive GENes for activity inference) can infer pathway activity for 14 signaling pathways (androgen, estrogen, EGFR, hypoxia, JAK‐STAT, MAPK, NFkB, PI3K, p53, TGFβ, TNFα, TRAIL, VEGF, and WNT) from gene expression data [17]. The progeny() function was used to calculate the activity score for each pathway in mouse scRNA‐seq data, thereby exploring which pathways play critical roles.

The decoupleR package [18] was used to obtain human transcription factor target gene regulatory networks via the get_collectri() function. Then, the activity of each transcription factor was calculated in the bulk transcriptomic data.

### 2.6 Biological Enrichment Analysis and Cell–Cell Interaction Analysis

This study integrated Gene Ontology (GO), Kyoto Encyclopedia of Genes and Genomes (KEGG), WikiPathways (https://www.wikipathways.org/), and Hallmark gene sets from the MSigDB database (http://www.gsea‐msigdb.org/gsea/msigdb/) to discover the underlying biological function. A ranked list of genes, based on their differential expression analysis, was subjected to Gene Set Enrichment Analysis (GSEA).

Understanding the complex interactions between cell types within the bone marrow microenvironment is crucial for elucidating the mechanisms of bone regeneration. Thus, CellChatDB was employed to explore interactions among different cell subpopulations.

### 2.7 Mice

C57BL/6 mice and CGRP−/− mice were used in this study. The C57BL/6 mice were purchased from Henan Skobes Biotechnology, and CGRP−/− mice were donated by Professor Guo Ren's research group at the School of Pharmacy, Central South University, after initial purchase from Cyagen Biotechnology. The mice used in this study were all 3‐month‐old males. The animal model for bone defects was established as previously reported [19, 20]. After the mice were anesthetized by intraperitoneal injection of 3% sodium pentobarbital, the hair at the knee joint was removed, the skin was cut, and the knee ligament was exposed. A hole was drilled in the intercondylar notch of the femur with a small drill bit, and a 0.6 mm diameter Kirschner needle was placed in the femur. All mice were euthanized one week after surgery, and femora were collected for follow‐up experiments. The metaphyseal cancellous bone of the bone regeneration area, located under the growth plate, indicated successful modeling. The animal model was evaluated by micro‐CT analysis, and only data from animals in which the model was successfully established were included in the analysis. All animal care procedures and experiments were examined and approved by the Animal Care and Use Committee of the Experimental Animal Research Center of the Second Xiangya Hospital of Central South University.

### 2.8 CGRP Configuration and Storage

The CGRP recombinant proteins were synthesized from Hongxun Biotechnology Co., Ltd., with the amino acid sequence: ACDTATCVTHRLAGLLSRSGGVVKNNFVPTNVGSKAF, 98% purity, length 37 aa. CGRP lyophilized powder was stored at −80°C, protein and resolubilization buffer PBS were removed and equilibrated to room temperature. After a short centrifugation, the lyophilized powder was collected at the bottom of the tube, and 1 mL PBS was added to 20 µg CGRP to prepare the mother liquor. After redissolving at 4°C for 15 min using a 360° shaker, aliquots were dispensed as needed and stored at −20°C.

### 2.9 Tube Formation Assay

The tube formation assay was conducted as previously reported [21]. After thawing the matrix on ice, 100 µL of the matrix was applied to a 96‐well plate and incubated at 37°C for 30 min to allow the matrix solution to solidify. Subsequently, a suspension of human microvascular endothelial cells (HMEC‐1, Research Resource Identifiers: CVCL_0307, Shanghai, China) that had been hypoxic for 1 h was added to each well, and treated with different concentrations of CGRP (0, 1.0, 5.0, 10.0, 20.0, 50.0 nM). The plates were then incubated at 37°C for 4 and 8 h to observe tube formation by the HMEC‐1 cells. Quantitative analysis of the number of tube branches and segments was performed by randomly selecting 4 microscopic fields per well.

### 2.10 Migration Assay

Transwell assay was employed to analyze the impact of different concentrations of CGRP on cell migration. Following serum starvation for 2 h, an HMEC‐1 suspension (1 × 10^5 cells/well) was seeded in the upper chamber of a 24‐well Transwell plate and co‐cultured with various concentrations of CGRP (0, 1.0, 5.0, 10.0, 20.0, 50.0 nM). The filter membrane had pores of 8 µm in diameter. After 24 h of incubation, the cells adhering to the upper surface of the filter membrane were gently wiped off using a cotton swab, while the cells that had migrated to the lower surface of the membrane were fixed in 4% paraformaldehyde for 30 min and subsequently stained with 0.5% crystal violet. Four microscopic fields were randomly selected from each well to quantify the number of migrated cells.

### 2.11 RT‐qPCR Analysis

Total RNA from CGRP‐treated HMEC‐1 was extracted using TRIzol reagent (Takara, 9108) and reverse‐transcribed into cDNA using the HiScript II 1st Strand cDNA Synthesis Kit (+gDNA wiper) (Vazyme, R212‐01). Real‐time PCR analysis was performed with SYBR Green (Vazyme, Q321‐02) in an Applied Biosystems QuantStudio 3 Real‐Time PCR System (Applied Biosystems), and the relative mRNA expression levels were normalized to endogenous β‐actin expression. The primer sequences are listed in Table 2.

**TABLE 3:** Baseline characteristics of 24 elderly subjects.

Number	Age(year)	Gender	Bone mineral density(g/cm2)	T‐value	WHO classification
1	73	male	1.265	0.7	normal
2	86	male	1.188	0.2	normal
3	65	male	1.265	0.7	normal
4	75	female	1.302	1.0	normal
5	63	male	1.093	−0.7	normal
6	68	female	1.120	−0.5	normal
7	66	female	1.136	−0.4	normal
8	75	male	1.196	0.2	normal
9	60	female	1.036	−1.2	low bone mass
10	91	female	1.020	−1.3	low bone mass
11	73	male	0.932	−2.1	low bone mass
12	70	female	0.945	−1.8	low bone mass
13	66	female	1.030	−1.3	low bone mass
14	60	male	0.948	−1.9	low bone mass
15	84	female	1.003	−1.4	low bone mass
16	69	male	0.976	−1.7	low bone mass
17	71	male	0.760	−3.5	osteoporosis
18	89	female	0.844	−2.8	osteoporosis
19	92	female	0.808	−3.1	osteoporosis
20	72	male	0.865	−2.8	osteoporosis
21	62	female	0.768	−3.4	osteoporosis
22	71	male	0.826	−2.9	osteoporosis
23	76	male	0.818	−3.0	osteoporosis
24	63	male	0.839	−2.8	osteoporosis

### 2.12 Western Blotting

Western blotting was performed as described previously [22]. Protein extracts were obtained by lysing cells on ice with RIPA buffer containing protease inhibitor for 30 min and then centrifuging at 4°C/12 000 rpm for 10 min. The protein concentrations were detected by bicinchoninic acid assay. The protein extracts were mixed with 5× sodium dodecyl sulfate (SDS)‐polyacrylamide gel electrophoresis (PAGE) loading buffer. The protein was denatured by boiling for 10 min at 100°C, and then separated by SDS‐PAGE and transferred to polyvinylidene fluoride membranes (Millipore). The membranes were then blocked with 5% milk and incubated overnight with primary antibodies against p‐FAK (Tyr397) (Cell Signaling Technology, 3283), p‐FAK (Tyr576/577) (Cell Signaling Technology, 3281), p‐FAK (Tyr925) (Cell Signaling Technology, 3284), FAK (Cell Signaling Technology, 3285), p‐AKT (Ser473) (Cell Signaling Technology, 4060), AKT (Cell Signaling Technology, 9272), p‐ERK (Thr202/Tyr204) (Cell Signaling Technology, 9101), ERK (Cell Signaling Technology, 9102), VEGFA (Proteintech, 19003‐1‐AP), OPN (Proteintech, 22952‐1‐AP), BMP2 (Abcam, ab214821), Runx2 (Abcam, ab214821), β‐actin (Cell Signaling Technology, 4967), CGRP (Cell Signaling Technology, 14 959), Angptl4 (Proteintech, 18374‐1‐AP), Alpl (Proteintech, 11187‐1‐AP), Ocn (Proteintech, 16157‐1‐AP), Spp1 (Proteintech, 22952‐1‐AP), Sp7 (Proteintech, 28694‐1‐AP), Postn (Proteintech, 66491‐1‐Ig), Fabp4 (Proteintech, 12802‐1‐AP), Pparg (Proteintech, 16643‐1‐AP), Lpl (Proteintech, 28602‐1‐AP), Plin1 (Proteintech, 83905‐4‐RR), Adipoq (Proteintech, 83961‐4‐RR), Id4 (Proteintech, 21803‐1‐AP), GAPDH (Proteintech, 60004‐1‐Ig), and Tubulin (Cell Signaling Technology, 2146) at 4°C. The membranes were then washed three times with PBST for 10 min each time. Secondary antibodies were added, incubated at room temperature for 1 h on a shaker, and washed three more times with PBST. Blots were performed with the use of enhanced chemiluminescence reagents (Bioprimacy, PMK0448). Quantitative analysis of optical density was performed using Image‐Pro Plus 6.0.

### 2.13 ELISA

Peripheral blood from the participants was left to separate before centrifugation, and the upper serum layer was collected. The serum was analyzed using the human CGRP ELISA kit (AiFang Biological, AF0833‐A). HMEC‐1 were treated with 50 ng/mL CGRP for 24 h, and then the supernatants were collected, and the secretion levels of Angptl4 were detected with an Angptl4 ELISA kit (AiFang Biological, AF1752‐A). All ELISAs were performed according to the manufacturer's instructions.

### 2.14 Phosphoproteome Analysis and Bioinformatic Analysis

Quantitative proteomic and bioinformatic analysis of TMT‐labeled phosphorylation was performed as described earlier [23]. Briefly, protein extraction was carried out with a standard protocol. The protein was enzymolized overnight with trypsin, and then the resultant hydrolyzed peptides were desalted with Strata X C18 (Phenomenex) and freeze‐dried in a vacuum. The peptides were labeled according to the instructions of the TMT kit. Then, they were separated by high pH reversed‐phase liquid chromatography on an Agilent 300Extend C18 column (5 µm particle diameter, 4.6 mm column diameter, 250 mm column length). Next, the peptides were dissolved in the enrichment buffer solution and eluted with 10% ammonia. The eluent was collected and desalted according to the C18 ZipTips instructions.

The peptides were dissolved in liquid chromatography mobile phase A and separated using the EASY‐nLC 1200 ultra‐high‐performance liquid system. Mobile phase A was an aqueous solution containing 0.1% formic acid and 2% acetonitrile. Mobile phase B was an aqueous solution containing 0.1% formic acid and 90% acetonitrile, with a gradient as follows: 0 to 38 min, 6% to 22% B; 38 to 52 min, 22% to 32% B; 52 to 56 min, 32% to 80% B; and 50 to 60 min, 80% B. The flow rate was kept at 500 µL/min. The samples were analyzed by QExactive HF‐X mass spectrometry. The differentially expressed proteins were further analyzed using the UniProt‐GOA database. Identified protein domain functional descriptions were annotated with InterProScan (a sequence analysis application) based on the protein sequence alignment method, and the InterPro domain database was used. Signaling pathway analysis was performed using the KEGG pathway database, and protein–protein interactions were searched through the STRING database.

### 2.15 Micro‐CT Analysis

Femora were extracted from all experimental groups of mice. After thorough removal of the attached soft tissue, the femora were fixed in 4% paraformaldehyde for 24 h. The fixed samples were scanned using high‐resolution micro‐CT (Skyscan 1172, Bruker microCT, Germany). The basic parameters of the scanner included an X‐ray energy tube voltage of 65 kV, a current of 153 µA, and a resolution of 15 µm per pixel. After scanning, image reconstruction software (NRecon, version 1.6, Bioz, USA), data analysis software (CT Analyser, version 1.9, Bruker microCT, Germany), and three‐dimensional model visualization software (µCT Volume, version 2.0, Bruker micro‐CT, Germany) were used to analyze the parameters of the distal femoral metaphyseal trabecular bone and generate a series of planar cross‐section images. We selected 5% of the femoral length below the growth plate for microarchitecture analysis. The following parameters were calculated to describe the subchondral trabecular bone microarchitecture: trabecular bone volume per tissue volume, trabecular number, trabecular separation, and trabecular thickness [24, 25].

### 2.16 Immunofluorescence and Immunocytochemistry of Bone Sections

The freshly dissected femora were fixed in 4% paraformaldehyde solution for 24 h, then decalcified in 0.5 M EDTA (pH 7.4) at 4°C for 21 d. Subsequently, dehydration was performed consecutively in 75%, 85%, 90%, 95%, and 100% alcohol, as well as xylene. The bone tissue was then embedded in embedding solution (8% gelatin, 2% polyvinylpyrrolidone, and 20% sucrose) and placed in a −80°C freezer overnight. Thereafter, the samples were longitudinally positioned and sectioned to a thickness of 15 µm. Staining was carried out using primary antibodies against CD31 (Abcam, ab182981, 1:500), EMCN (Abcam, ab106100, 1:200), VEGFA (Boster Bio, BA0407, 1:200), Runx2 (Abcam, ab192256, 1:500), CGRP (Cell Signaling Technology, #14 959, 1:800), PGP9.5 (Proteintech, 14730‐1‐AP, 1:800), LEPR (Proteintech, 20966‐1‐AP, 1:50), and ANGPTL4 (Proteintech, 18374‐1‐AP, 1:500). The samples were incubated overnight at 4°C. On the second day, the samples were incubated with fluorescently labeled secondary antibodies for 60 min at room temperature, followed by incubation with DAPI (BL105A, Biosharp) working solution for 5 min. After washing three times with PBS, the tissue was treated with antifading mounting medium, a coverslip was placed, and fluorescent signals were captured using a fluorescence microscope. Additionally, the bone tissue was embedded in paraffin, sectioned to 4 µm, and subjected to immunohistochemical staining. The paraffin sections were dewaxed, and antigen retrieval was performed using 2× citric acid (pH 6.0). Staining was then carried out using the primary antibody against Ocn, followed by visualization with DAB (Proteintech, PR30010) working solution, counterstaining with hematoxylin, dehydration, and mounting.

### 2.17 Flow Cytometric Analysis

Mouse femora and tibiae were isolated, and their metaphyseal regions were crushed in ice‐cold PBS. The cells were digested with 1 mg/mL collagenase type II (Sigma, V900892) for 30 min at 37°C. After filtration and washing, red blood cell lysis buffer (Solarbio, R1010) was added for 5 min, and the suspension was shaken before filtering through a 100 µm cell filter to obtain a single‐cell suspension. After blocking the cells with 0.5% bovine serum albumin for 15 min, they were incubated with APC‐labeled anti‐CD31 antibody (BD Biosciences, 551 262) and PE‐labeled anti‐Emcn antibody (Thermo Fisher, 12‐5851‐82) for 45 min at 4°C and washed 1‐2 times with 0.5% bovine serum albumin and 2 mM EDTA to obtain CD31highEmcnhigh cells. Finally, data were obtained on a BD FACScan cytometer. We analyzed cell populations with high expression of CD31 and EMCN.

### 2.18 Statistical Analyses

The single‐cell and bulk RNA‐seq datasets in this study were processed using R software (version 4.4.0). Statistical analysis was conducted using ImageJ and GraphPad Prism 9 software. Micro‐CT images were analyzed for bone histomorphometric parameters using CTAn, CTVol, and DataViewer. All data were expressed as the mean ± standard deviation. The *t*‐test was used to compare the means between two groups, whereas the one‐way analysis of variance was employed to compare the means among multiple groups. Pearson's correlation coefficient was used to assess correlations between two continuous variables, while unpaired t‐tests or Wilcoxon rank‐sum tests were employed to compare gene expression or signature scores across groups. Statistical significance was set at *p < 0.05*, indicated by “^*^,” “^**^” for *p < 0.05*, “^***^” for *p < 0.01*, and “^****^” for *p < 0.001*.

## 3 Results

### 3.1 Expression of CGRP in Patients with Osteoporosis

Initially, serum CGRP levels in patients with osteoporosis were evaluated. The demographic characteristics of the individuals in this study are shown in Tables 1 and 3. The participants were from the Second Xiangya Hospital of Central South University. Among them, eight patients <60 years old with normal bone mass were included in the young healthy control group, eight patients >60 years old with normal bone mass were included in the elderly healthy control group, eight patients >60 years old with low bone mass were included in the osteopenia group, and eight patients >60 years old with osteoporosis were included in the osteoporosis group. Fasting peripheral blood serum was collected from each group in the morning, and the serum level of CGRP was determined by enzyme‐linked immunosorbent assay (ELISA).

**TABLE 2:** Primer sequence.

Gene	Forward Primer(5′‐3′)	Reverse Primer(5′‐3′)
ANGPTL4	GGCTCAGTGGACTTCAACCG	CCGTGATGCTATGCACCTTC
GAPDH	GGAGCGAGATCCCTCCAAAAT	GGCTGTTGTCATACTTCTCATGG
RUNX2	ATGCTTCATTCGCCTCACAAA	GCACTCACTGACTCGGTTGG
ALP	CCCCATGTGATGGCGTAT	CGGTAGGGAGAGCACAGC
Osterix	ATGGCGTCCTCTCTGCTTG	TGAAAGGTCAGCGTATGGCTT
POSTN	CAGCAAACCACTTTCACCGACC	AGAAGGCGTTGGTCCATGCTCA
OPN	ACCATGCAGAGAGCGAGGATT	GGGACATCGACTGTAGGGACG
OCN	AAGCAGGAGGGCAATAAGGT	ATGCGTTTGTAGGCGGTCTT
FABP4	AAGGTGAAGAGCATCATAACCCT	TCACGCCTTTCATAACACATTCC
PPARγ	ATGGTTGACACAGAGATGC	GAATGCGAGTGGTCTTCC
Perilipin	GAGAAGGTGGTAGAGTTCCTCC	GTGTGTCGAGAAAGAGTGTTGGC
ID4	CAGTGCGATATGAACGACTGC	GACTTTCTTGTTGGGCGGGAT
LPL	AAGAAGCAGCAAAATGTACCTGAAG	CCTGATTGGTATGGGTTTCACTC
Adipoq	TGTTCCTCTTAATCCTGCCCA	CCAACCTGCACAAGTTCCCTT
β‐actin	GGCTGTATTCCCCTCCATCG	CCAGTTGGTAACAATGCCATGT

**FIGURE 2:**
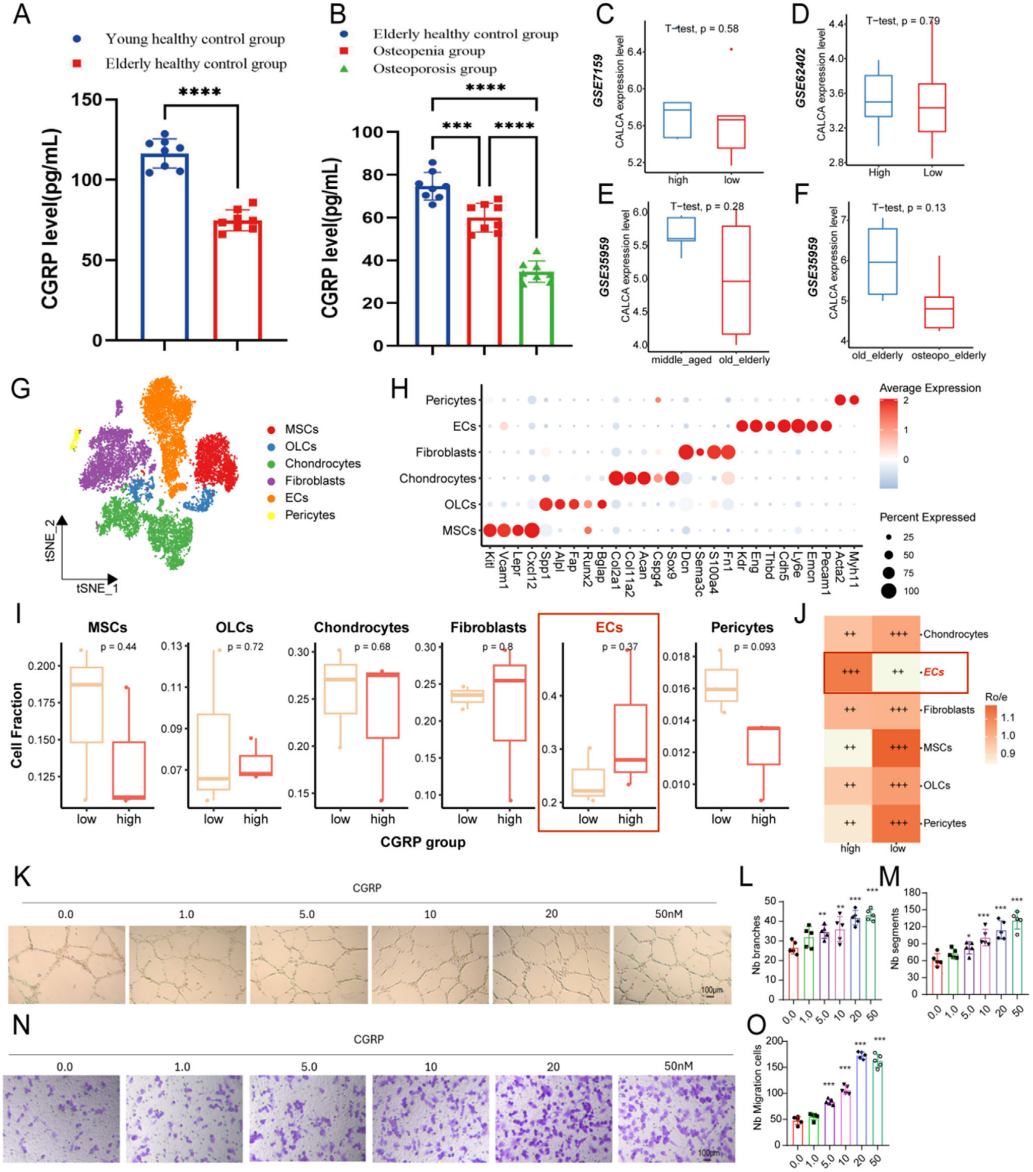
Expression of CGRP in patients and CGRP exhibited pro‐angiogenic effects on endothelial cells. (A) Analysis of CGRP expression levels in eight young healthy individuals and eight elderly healthy individuals (Student's *t*‐test). (B) Analysis of CGRP expression levels among elderly healthy individuals, elderly individuals with low bone mass, and elderly individuals with osteoporosis (one‐way analysis of variance). ^***^ represents *p* = 0.0003, and ^****^ represents *p* < 0.001. (C and D) CGRP expression in patients with high and low BMD in (C) GSE7159, (D) GSE6240. (E) CGRP expression in patients with middle‐aged and elderly patients in GSE35959. (F) CGRP expression in patients with non‐osteoporotic and osteoporotic elderly patients in GSE35959. (G) T‐SNE of mouse scRNA‐seq. (H) Markers genes in MSCs, OLCs, Chondrocytes, Fibroblasts, ECs, and Pericytes. (I) Comparison of various cell types between the high‐ and low‐CGRP group. (J) Ro/e analysis of various cell types between high‐ and low‐CGRP group. (K–M) Representative images (K) and relative quantification of tube branch numbers (L) and tube segments (M) in a Matrigel tube formation assay. Scale bar, 100 µm. (N‐O) Representative images (N) and relative quantification of migrated cells (O) by transwell migration assay. The data are shown as the mean ± standard deviation. ^*^ *p* < 0.05, ^**^ *p* < 0.01 and ^***^ *p* < 0.001 by one‐way analysis of variance (L,M, and O).

Elderly individuals 1–8 comprised the healthy group, 9–16 were the low bone mass group, and 17–24 were the osteoporosis group (Table 3). The young healthy group and the elderly healthy group were compared, and found that the CGRP level was significantly lower with older age, and the result was not affected by sex (Figure 2A). Upon comparing results for the elderly normal, low bone mass, and osteoporosis groups, we found that bone mass and CGRP levels were lower in individuals with lower bone mineral density (Figure 2B). These findings suggest that CGRP level in human serum is inversely proportional to age and directly proportional to bone mass, serving as a potential marker for predicting osteoporosis.

Patients with high BMD exhibited higher CGRP (CALCA gene) expression in GSE7159 (Figure 2C), GSE6240 (Figure 2D), GSE56814 (Figure S1A), and GSE56815 datasets (Figure S1B), and middle‐aged patients demonstrated greater CGRP expression than elderly patient in GSE35959 datasets (Figure 2E). Furthermore, GSE35959 datasets revealed that non‐osteoporotic elderly patients had higher CGRP levels compared to osteoporotic elderly patients (Figure 2F). Although statistical significance was not achieved, likely due to sample size limitations, thes resulte trends further corroborate our findings.

### 3.2 CGRP Exhibited Pro‐Angiogenic Effects on Endothelial Cells

In mouse scRNA‐seq, T‐SNE dimensionality reduction resolved seven major cell clusters, including MSCs, osteolineage cells (OLCs), Chondrocytes, Fibroblasts, endothelial cells (ECs), and Pericytes (Figure 2G). Figure 2H presents the expression of significant markers in all cell types. Interestingly, high‐ and low‐ CGRP groups were defined based on Calca1 (encoding CGRP) expression levels in pseudobulk data, revealing ECs enrichment in the high CGRP group (Figure 2I). Moreover, Ro/e analysis also confirmed ECs enrichment in the high CGRP group (Figure 2J), indicating a tight association between CGRP and ECs. To further investigate the effect of CGRP on ECs, HMECs were treated with complete medium containing varying concentrations of CGRP (0.0, 1.0, 5.0, 10.0, 20.0, or 50.0 nM) for a tube formation assay. The tube formation assay on Matrigel, a rapid, quantifiable method for measuring angiogenesis in vitro, revealed that HMEC‐1 cells treated with CGRP formed more vascular‐like structures than negative controls, and that CGRP promoted angiogenesis in a dose‐dependent manner (Figure 2K). The quantitative results showed that CGRP treatment increased the number of tube branches and segments (Figure 2L,M). In addition, transwell assays revealed that CGRP promoted the migration of HMEC‐1 cells and significantly increased the number of migrating cells. The migration‐promoting ability of CGRP was the strongest at a concentration of 20.0 nM (Figure 2N,O). Taken together, CGRP could promote angiogenesis by HMEC‐1 in a dose‐dependent manner.

### 3.3 CGRP Supported Endothelial Angiogenesis Through the FAK–AKT–VEGF Signaling Pathway

**FIGURE 3:**
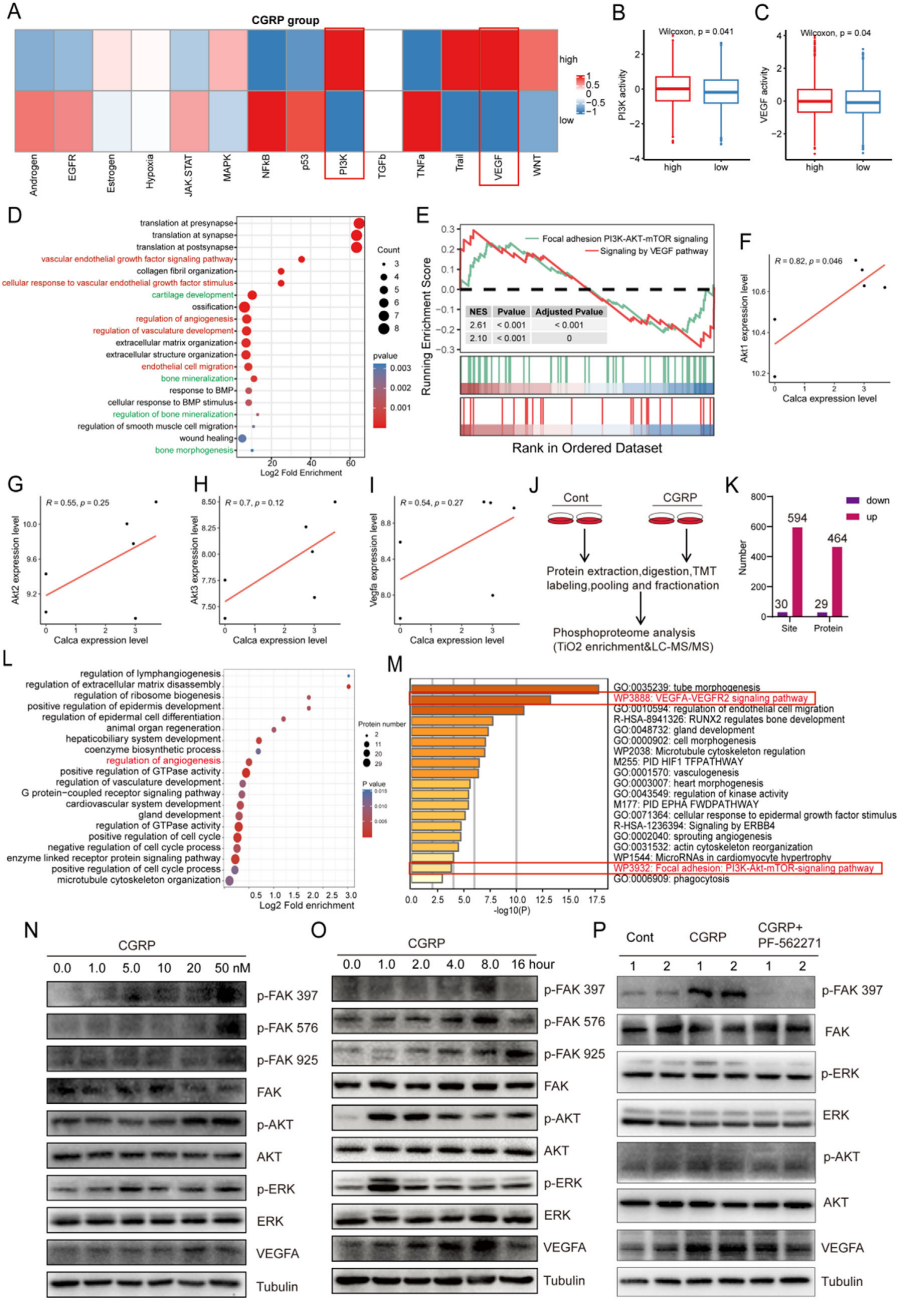
CGRP promotes endothelial angiogenesis by activating the FAK–AKT–VEGF signaling pathway. (A) The activity score for 14 signaling pathways (androgen, estrogen, EGFR, hypoxia, JAK‐STAT, MAPK, NFkβ, PI3K, p53, TGFβ, TNFα, TRAIL, VEGF, and WNT) in scRNA‐seq data. (B) PI3K pathway activity in high‐ and low‐CGRP groups. (C) VEGF pathway activity in high‐ and low‐CGRP groups. (D) Enrichment analysis of differentially expressed genes between high and low CGRP groups. (E) GSEA results of the PI3K‐AKT‐mTOR and VEGF signaling pathways. (F–I) Correlation analysis between CGRP expression and Akt1/Akt2/Akt3/Vegfa expression. (J) Experimental workflow for TMT labeling‐based phosphoproteomic analysis of HMEC‐1. (K) Quantitative analysis of phosphorylation sites and phosphoproteins identified by liquid chromatography–mass spectrometry. (L) GO enrichment analysis of common up‐regulated and down‐regulated phosphoproteins using the package clusterProfiler. The top 20 GO terms are displayed. (M) Kinase enrichment analysis was performed. The enriched kinases are depicted as a bubble plot. (N) Western blotting of p‐FAK 397, p‐FAK 576, p‐FAK 925, FAK, p‐AKT, AKT, p‐ERK, ERK, VEGFA, and tubulin in HMEC‐1 cells treated with different concentrations of CGRP. (O) Western blotting of p‐FAK 397, p‐FAK 576, p‐FAK 925, FAK, p‐AKT, AKT, p‐ERK, ERK, VEGFA, and tubulin at different time points in HMEC‐1 cells treated with the same concentration of CGRP. (P) Western blotting of p‐FAK 397, FAK, p‐ERK, ERK, p‐AKT, AKT, VEGFA, and tubulin in HMEC‐1 cells treated with Control group, CGRP group, and FAK inhibitors (PF‐562271) group.

Next, to explore the specific molecular mechanism by which CGRP affects angiogenesis in HMECs, PROGENy was performed to calculate the activity score for 14 signaling pathways in scRNA‐seq data. Pathway activity analysis revealed elevated signaling in the P13K and VEGF pathways within the high CGRP group (Figure 3A–C), while most other signaling pathways exhibited higher expression in the low CGRP group (Figure S1C–N). Enrichment analysis of differentially expressed genes between high and low CGRP groups revealed enrichment in biological processes related to angiogenesis and bone differentiation, such as vascular endothelial growth factor signaling pathway, cellular response to vascular endothelial growth factor stimulus, regulation of angiogenesis, regulation of vasculature development, bone mineralization, and regulation of bone mineralization (Figure 3D). GSEA results further revealed enrichment of the PI3K‐AKT‐mTOR and VEGF signaling pathways (Figure 3E). Subsequently, this study further explored the correlations between CGRP and Akt/VEGF. Correlation analysis revealed that as CGRP expression increased, the expression of Akt1/2/3 and Vegfa also increased (Figure 3F–I), showing significant correlation. The same trend in results is observed in GSE7159 (Figure S2A), GSE35959 (Figure S2B), GSE56814 (Figure S2C), GSE56815 (Figure S2D), and GSE6240 datasets (Figure S2E). These results suggest that CGRP may promote endothelial angiogenesis through AKT and VEGF signaling pathways.

To validate our findings, we quantitatively analyzed phosphorylation between the CGRP‐treated and control groups using TMT labeling (Figure 3J). After protein extraction, digestion, TMT labeling, pooling, and fractionation, TiO2 enrichment and liquid chromatography–tandem mass spectrometry (LC‐MS/MS) analysis were performed to obtain reliable identification results. A total of 624 differentially modified phosphorylation sites were identified by LC‐MS/MS, including 594 sites with greater phosphorylation and 30 with less phosphorylation, and 493 differentially modified phosphorylated proteins, including 464 proteins with greater phosphorylation and 29 with less phosphorylation (Figure 3K). We performed kinase enrichment analysis and visualized the top 20 most significantly enriched categories in the bubble plot, which include biological processes involved in regulating angiogenesis (Figure 3L). Next, the enrichment analysis from the differentially modified proteins results showed that molecules involved in tube morphogenesis, regulation of endothelial cell migration, and vasculogenesis were significantly enriched. The enrichment analysis also indicated that the AKT and VEGFA–VEGFR2 signaling pathways are closely related to the vasogenic effect of CGRP (Figure 3M). Then, via comparison with the STRING (v.11.0) protein interaction networks database, we constructed a graph of the differentially modified protein interaction relationships using the criterion confidence score>0.7 (high confidence). Kinase enrichment analysis showed that PTK2, NCK1, PKD1, and EPHA2 were highly closely related, and their expressions played an important role in angiogenesis, which may be the target genes of CGRP on HMEC‐1 (Figure S2A). The heatmap of the proteins associated with angiogenesis that were more phosphorylated showed that PTK2 was significantly more phosphorylated in the CGRP‐treated group than the control group (Figure S2B). Likewise, correlation analysis revealed a significant correlation between PTK2 and CGRP (Figures S2C,E–I). These results suggested that PTK2 (namely FAK) might be a key factor in the regulation of angiogenesis by CGRP. Of note, CGRP expression levels show a positive correlation with FAK‐PI3K‐AKT‐MTOR signaling activity (Figure S2D–I).

To further confirm the changes in protein expression shown by scRNA‐seq data, bulk transcriptomic data, and phosphorylated proteomics analysis, and to detect if CGRP promotes angiogenesis through this signaling pathway, HMEC‐1 cells were treated with 0.0, 1.0, 5.0, 10.0, 20.0, or 50.0 nM CGRP for 24 h. Protein levels of p‐focal adhesion kinase (p‐FAK) 397, p‐FAK 576, p‐FAK 925, FAK, p‐AKT, AKT, p‐ERK, ERK, VEGFA, and tubulin in HMEC‐1 were evaluated by western blotting. Blotting analysis revealed that, compared with the control group, the HMEC‐1 treated with CGRP had higher protein levels of p‐FAK 397, p‐FAK 576, p‐FAK 925, FAK, p‐AKT, p‐ERK, and VEGFA. The protein levels of FAK, AKT, and ERK were not significantly changed, suggesting that CGRP could induce the phosphorylation, rather than production, of FAK, AKT, and ERK (Figure 3N). Then, after HMEC‐1 cells were treated with the same concentration of CGRP for 0, 1, 2, 4, 8, and 16 h, western blotting was used to further evaluate the expression levels of the proteins: expression levels of p‐FAK 397, p‐FAK 576, and VEGFA were the highest after 8 h of treatment; p‐FAK 925 was highest after 16 h of treatment; and p‐AKT and p‐ERK were highest after 1–2 h of treatment. However, the expression of FAK, AKT, and ERK did not change with time (Figure 3O). Notably, the addition of FAK inhibitors (PF‐56227, the first compound to enter clinical studies, which inhibits phosphorylation of the Y397 residue of FAK in a structure‐ and dose‐dependent manner) to the CGRP group resulted in varying degrees of suppression in the expression levels of p‐ERK, ERK, p‐AKT, AKT, and VEGFA (Figure 3P). Therefore, the FAK–AKT–VEGF signaling pathway may be the potential mechanism by which CGRP promotes endothelial angiogenesis.

### 3.4 CGRP−/−Knockout Mice had Lower Bone Vascularity and Bone Mass than WT Mice

**FIGURE 4:**
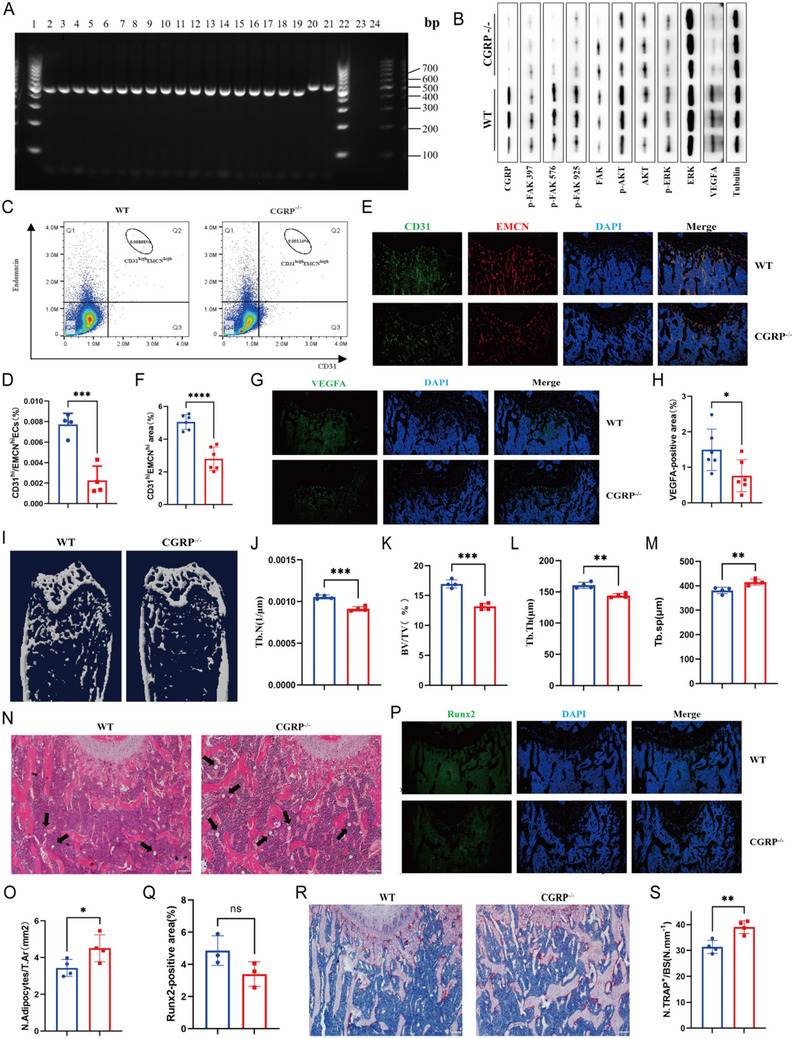
Cgrp^−^/^−^ mice exhibited reduced bone angiogenesis and lower bone formation, and bone mass than WT controls. (A) Agarose gel electrophoresis images of samples from wild type (WT) and Cgrp knockout (Cgrp−/−) mice. (B) Western blotting of CGRP, p‐FAK 397, p‐FAK 576, p‐FAK 925, FAK, p‐AKT, AKT, p‐ERK, ERK, VEGFA, and tubulin in HMEC‐1 cells with WT mice and CGRP−/− knockout mice. (C and D) Flow cytometry dot plot and quantification of CD31highEmcnhigh endothelial cells from WT and Cgrp−/− mice. (E and F) Representative images and quantification of CD31 (green)‐ and Emcn (red)‐stained femora from WT and Cgrp−/− mice. Scale bar, 100 µm. (G and H) Representative images and quantitation of Vefga (green) immunostaining in the femora of WT and Cgrp−/− mice. Scale bar, 100 µm. *n* = 3 mice in each group. (I–M) Representative micro‐CT images and quantitative micro‐CT analysis of the trabecular bone microarchitecture of WT and Cgrp−/− mice. Scale bar, 1 mm. (N and O) Representative images of hematoxylin–eosin staining in distal femora and quantification of the number of adipocytes related to the tissue area. Scale bar, 100 µm. (P and Q) Representative images and quantitation of Runx2 (green) immunostaining in femora of WT and Cgrp−/− mice. Scale bar, 100 µm. (R and S) Representative images of TRAP staining and quantification of TRAP‐positive cells on the trabecular bone surfaces of WT and Cgrp−/− mice. Scale bar, 100 µm. *n =* 3 mice in each group. The data are shown as the mean ± standard deviation. ^*^ *p* < 0.05; ^**^ *p* < 0.01; ^***^, and *p* < 0.001 by Student's *t*‐test and one‐way analysis of variance (J–M). BV/TV, trabecular bone volume per tissue volume; Tb. N, trabecular number; Tb. Sp, trabecular separation; Tb. Th, trabecular thickness.

Following our demonstration of the angiogenic effect and mechanism of CGRP in vitro, we next investigated whether CGRP regulates bone angiogenesis in vivo. We constructed CGRP−/− mice and analyzed the effects of CGRP deficiency on bone vasculature and phenotype. Agarose gel electrophoresis was used to determine the genotypes of the mice. Genomic DNA from mouse tails was amplified by polymerase chain reaction (PCR): a band of approximately 419 bp indicated a CGRP−/− mouse, and a band of approximately 479 bp indicated a WT mouse (Figure 4A). Western blot analysis revealed extremely low levels of CGRP expression in CGRP−/− knockout mice, confirming the successful construction of the CGRP−/− knockout mice model (Figures 4B and S3A,B). As expected, protein levels of p‐focal adhesion kinase (p‐FAK) 397, p‐FAK 576, p‐FAK 925, p‐AKT, p‐ERK, and VEGFA decreased in the knockout mice (Figures 4B and S3A,B). There was no significant difference in body weight between CGRP−/− and WT mice (Figure S3C–E). Flow cytometric sorting and quantitative analysis of H‐type endothelial cells showed that the number of CD31^high^Emcn^high^ endothelial cells in the bone marrow of 3‐month‐old CGRP−/− mice was lower than in 3‐month‐old WT mice (Figure 4C,D). CD31 and Emcn co‐immunostaining revealed that the number of H‐type vessels in CGRP−/− mice was significantly lower than that in age‐matched WT mice (Figure 4E,F). VEGFA is a key mediator in regulating angiogenesis, and VEGFA immunostaining demonstrated that the loss of CGRP reduces angiogenesis (Figure 4G,H).

H‐type blood vessels are associated with angiogenesis and osteogenesis. To explore if the absence of CGRP affects bone mass in vivo, we performed micro‐computed tomography (micro‐CT) and histomorphologic analysis on the femora of 3‐month‐old WT and CGRP−/− mice, which showed that CGRP−/− mice had an osteoporosis phenotype. Trabecular bone mass and density, trabecular thickness, trabecular number, and trabecular separation were lower in CGRP−/− than in WT mice (Figure 4I,M). Hematoxylin–eosin staining showed that the bone trabecular level in CGRP−/− mice was lower than that in WT mice (Figure 4N,O). Runx2 is involved in osteoblast differentiation and bone morphogenesis, and the expression of Runx2 is significantly lower in CGRP−/− mice than in WT counterparts (Figure 4P,Q). In addition, TRAP staining showed that CGRP−/− mice had significantly more TRAP‐positive osteoclasts than the control group (Figure 4R,S). These findings suggested that the loss of CGRP leads to impaired H‐type endothelial cell formation and bone formation, and promotes bone resorption.

### 3.5 CGRP−/−Mice had Lower Bone Vascularity and Delayed Bone Regeneration During Bone Defect Repair than WT Mice

**FIGURE 5:**
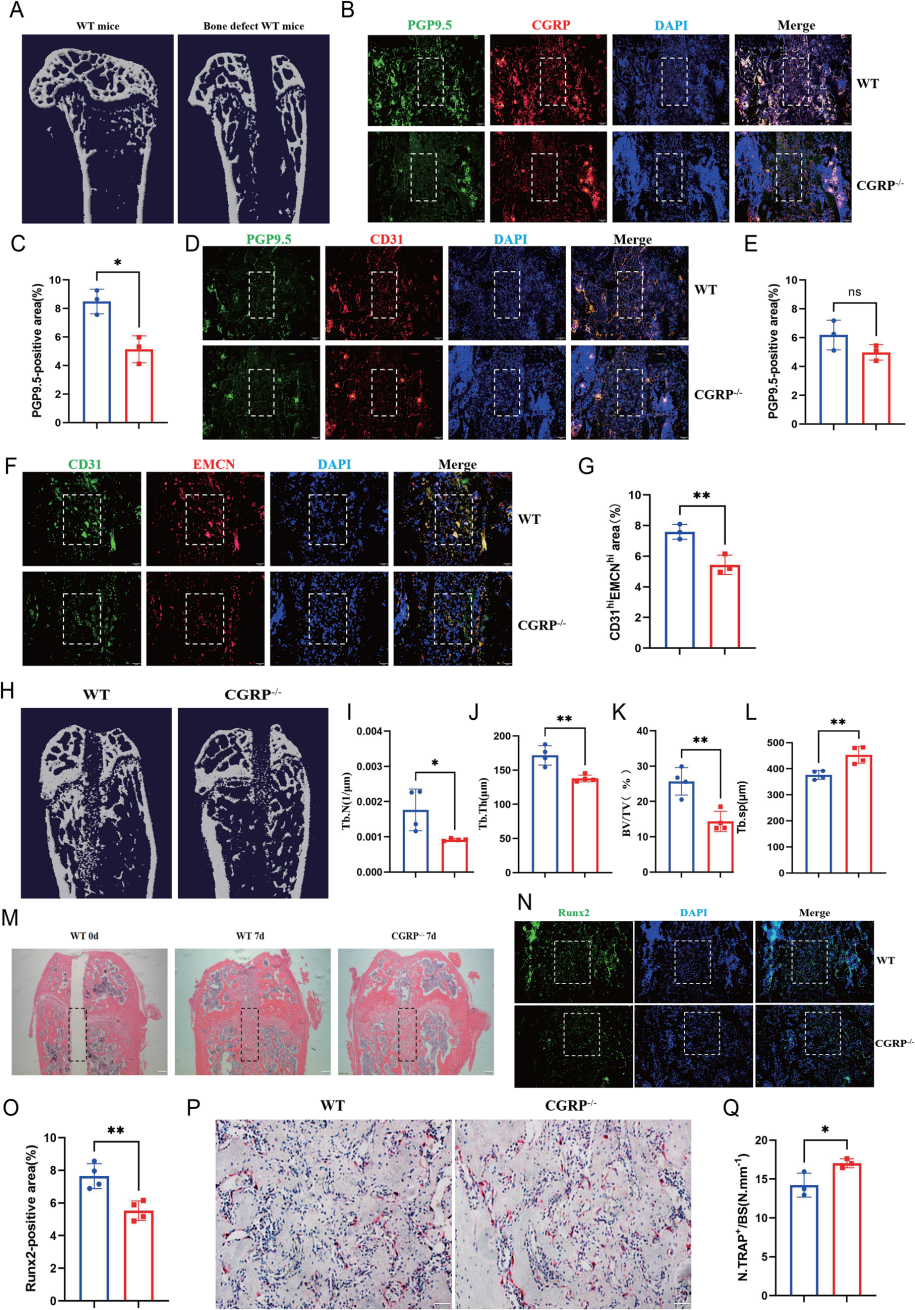
During bone defect repair, Cgrp^−^/^−^ mice had lower bone angiogenesis than WT mice and exhibited delayed bone regeneration. (A) Representative micro‐CT images of the trabecular microstructure of WT mice before and after bone defect modeling. (B and C) Representative images and quantification of PGP9.5 (green)‐ and CGRP (red)‐stained femora from WT and Cgrp−/− mice after bone defect introduction. Scale bar, 100 µm. (D and E) Representative images and quantification in PGP9.5 (green)‐ and CD31 (red)‐stained femora from WT and Cgrp−/− mice after bone defects. Scale bar, 100 µm. (F and G) Representative images and quantification of CD31 (green)‐ and Emcn (red)‐stained femora from WT and Cgrp−/− mice after bone defect introduction. Scale bar, 100 µm. *n* = 3 mice in each group. (H–L) Representative micro‐CT images and quantitative micro‐CT analysis of the trabecular bone microarchitecture of WT and Cgrp−/− mice after bone defect introduction. Scale bar, 1 mm. (M) Representative images of hematoxylin–eosin staining in the bone generation area of WT and Cgrp−/− mice. Scale bar, 200 µm. (N and O) Representative images and quantitation of Runx2 (green) immunostaining in the bone generation area of WT and CGRP−/− mice. Scale bar, 100 µm. (P‐Q) Representative images of TRAP staining and quantification of TRAP‐positive cells on the trabecular bone surfaces of WT and CGRP−/− mice. Scale bar, 100 µm. *n* = 3 mice in each group. The data are shown as the mean ± standard deviation. ^*^ *p* < 0.05; ^**^ *p* < 0.01; and ^***^ *p* < 0.001 by Student's *t*‐test. BV/TV, trabecular bone volume per tissue volume; Tb. N, trabecular number; Tb. Sp, trabecular separation; Tb. Th, trabecular thickness.

Next, we explored the relationship between H‐type angiogenesis after CGRP knockout and bone regeneration. The bone defect model was established by surgically removing the bone trabeculae of 3‐month‐old WT and CGRP−/− mice; the femora were removed for further treatment one week after the operation (Figure 5A).

Firstly, we observed the expression of PGP9.5 and CGRP in CGRP−/− mice after bone injury. The expression of PGP9.5 and CGRP was significantly lower in CGRP−/− mice, suggesting a coupled relationship between CGRP and neurogenesis‐related factor PGP9.5 (Figure 5B,C). The expression of PGP9.5 and CD31 indicated that bone injury repair promoted neurogenesis and angiogenesis, and further demonstrated a spatial coupling of the two (Figure 5D,E). Thereafter, we verified the effect of the absence of CGRP on vascular remodeling. Co‐immunostaining for CD31 and Emcn revealed that, one week post‐surgery, H‐type angiogenesis in the bone regeneration area of CGRP ‐/‐ mice was less abundant compared to the WT group 1 week post‐surgery (Figure 5F,G). To further assess whether the reduction in H‐type vessels caused by CGRP loss also delayed the bone regeneration process, we examined the bone regeneration phenotype. As expected, micro‐CT analysis and hematoxylin–eosin staining showed that CGRP−/− mice, compared with the control group, possessed less bone regeneration and a lower trabecular number in the bone defect area 1 week after surgery (Figure 5H–M). Runx2 immunostaining also exhibited significantly lower Runx2 expression in CGRP−/− mice (Figure 5N–O). Besides, in the bone regeneration area of CGRP−/− mice, the number of TRAP‐positive osteoclasts was higher than in WT mice (Figure 5P,Q). These data indicate that bone regeneration ability is closely related to the abundance of bone vessels at the injured site, and CGRP deficiency reduces the formation of bone vessels and delays bone regeneration.

### 3.6 Exogenous Recombinant CGRP Protein Therapy Can Promote Bone Regeneration After Bone Injury

**FIGURE 6:**
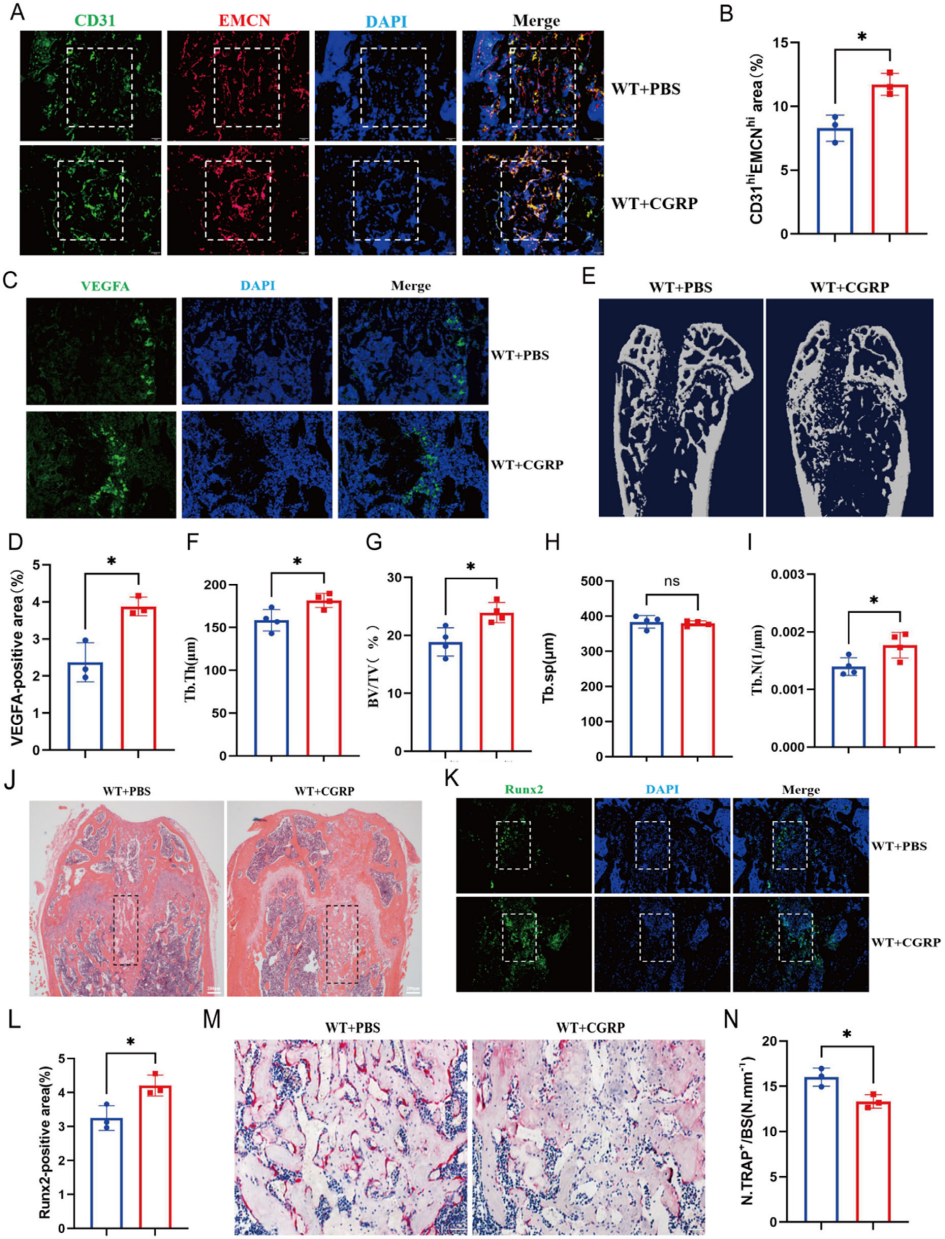
Recombinant Cgrp protein restored bone formation by promoting bone angiogenesis. (A and B) Representative images and quantification of CD31 (green)‐ and Emcn (red)‐stained femora from WT mice after bone defect introduction, and injection of the experimental group with recombinant Cgrp protein. Scale bar, 100 µm. (C and D) Representative images and quantitation of Vefga (green) immunostaining in the femora of WT and Cgrp−/− mice. Scale bar, 100 µm. (E‐I) Representative micro‐CT images and quantitative micro‐CT analysis of trabecular bone microarchitecture in WT mice after bone defect introduction, and injection of the experimental group with recombinant CGRP protein. Scale bar, 1 mm. (J) Representative images of hematoxylin–eosin staining in the bone generation areas of the PBS group and CGRP treatment group. (K and L) Representative images and quantitation of Runx2 (green) immunostaining in the bone generation areas of the phosphate‐buffered saline (PBS) group and CGRP treatment group. Scale bar, 100 µm. (M and N) Representative images of TRAP staining and quantification of TRAP‐positive cells on the trabecular bone surfaces of the PBS group and CGRP treatment group. Scale bar, 100 µm. *n* = 3 mice in each group. The data are shown as the mean ± standard deviation. ^*^ *p* < 0.05, ^**^ *p* < 0.01; and ^****^ *p* < 0.0001by Student's *t*‐test. BV/TV, trabecular bone volume per tissue volume; Tb. N, trabecular number; Tb. Sp, trabecular separation; Tb. Th, trabecular thickness.

After we demonstrated that the absence of CGRP can reduce the formation of bone vessels and delay the process of bone regeneration, we observed the effect of exogenous CGRP treatment on bone regeneration in bone defect mice. The bone defect model was employed in 3‐month‐old mice, and 10 mg/kg of recombinant CGRP protein was injected into 3‐month‐old WT bone defect mice through the tail vein on the 1st, 3rd, and 5th day after surgery. The control group was injected with the same amount of phosphate‐buffered saline (PBS) solution via the same protocol, and the femora of the mice were removed on the 7th day for follow‐up analysis. After CGRP treatment, the CD31^high^Emcn^high^ areas of endothelial cells and the VEGFA‐positive areas were larger than those in the control group, indicating that CGRP could promote the formation of H‐type blood vessels in the bone regeneration area (Figure 6A–D). Micro‐CT analysis and histomorphometric analysis revealed that CGRP injection had a more obvious effect on bone repair in the area of the bone defect (Figure 6E–I). Hematoxylin–eosin staining showed that the trabecular bone level in the CGRP treatment group was higher than that in the control group (Figure 6J). After CGRP treatment, the number of Runx2‐positive cells in the femoral metaphyses of the treatment group was higher than that of the control group, but the treatment had the opposite effect on the number of osteoclasts (Figure 6K–N). These results indicated that exogenous CGRP treatment can promote angiogenesis and bone repair in the bone regeneration area of mice with bone defects.

### 3.7 CGRP Regulates the Secretion of ANGPTL4 by Vascular Endothelial Cells

**FIGURE 7:**
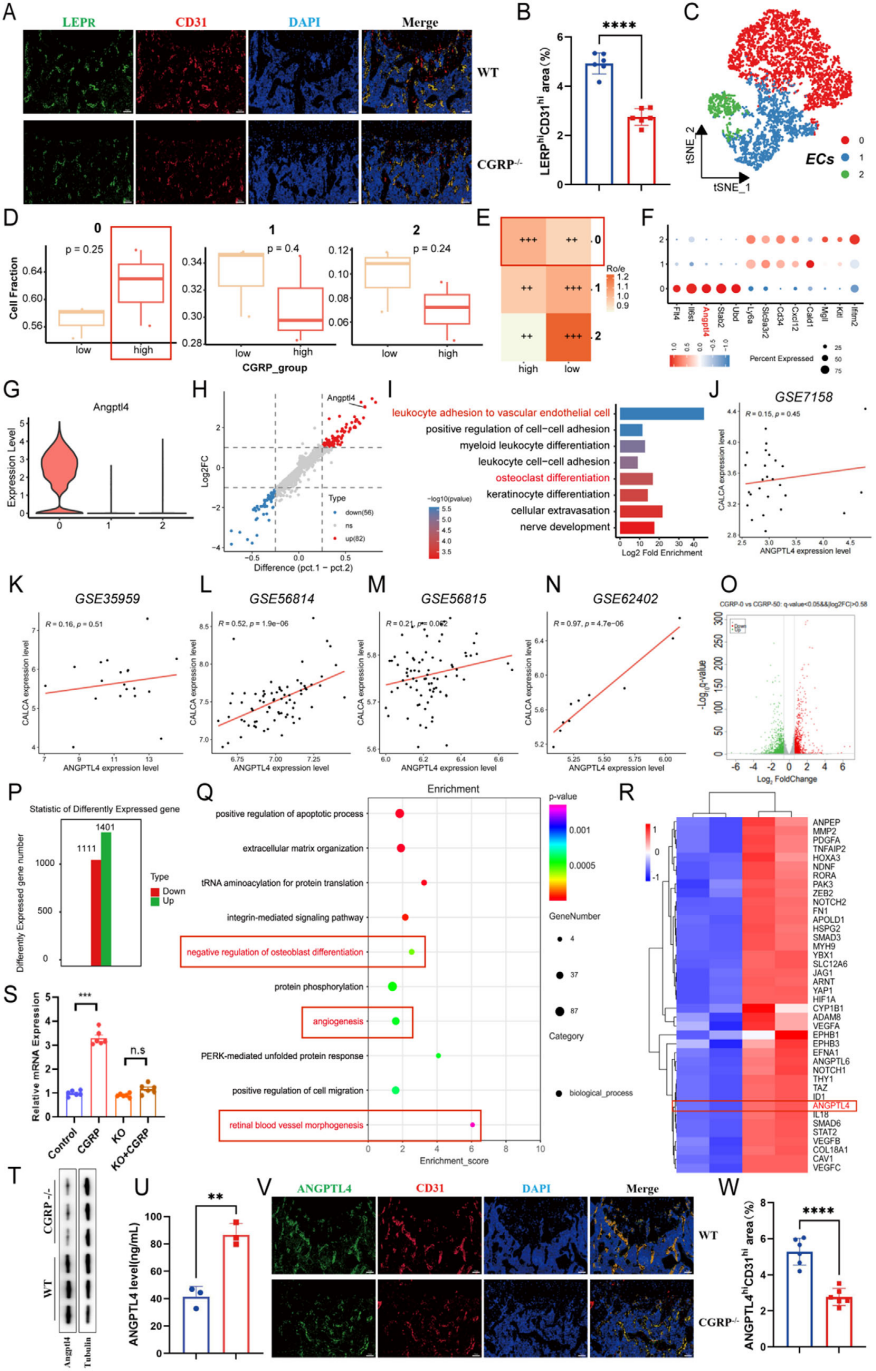
CGRP regulates the secretion of ANGPTL4 by vascular endothelial cells. (A‐B) Representative images and quantification of Lepr (green)‐ and CD31 (red)‐stained femora from WT and CGRP−/− mice. Scale bar, 100 µm. (C) T‐SNE of ECs in mouse scRNA‐seq. (D) Comparison of ECs cluster 0/1/2 between high‐ and low‐CGRP group. (E) Ro/e analysis of ECs cluster 0/1/2 between high‐ and low‐CGRP group. (F) Markers genes in ECs cluster 0/1/2. (G) Expression of the Angptl4 gene in ECs cluster 0/1/2. (H) Volcano plot of differentially expressed genes between ECs cluster 0 and ECs cluster 1/2. (I) Enrichment analysis of differentially expressed genes between ECs cluster 0 and ECs cluster 1/2. (J–N) Correlation analysis between ANGPTL4 gene expression and CGRP gene expression in (J) GSE7158, (K) GSE35959, (L) GSE56814, (M) GSE56815, and (N) GSE62402. (O and P) Number of differentially expressed genes upon treatment with CGRP. The red bar represents up‐regulated genes. The green bar represents down‐regulated genes. (Q) GO enrichment analysis of common up‐regulated and down‐regulated genes using the package clusterProfiler. The top 10 GO terms are displayed. (R) Fold change (log2) expression of up‐regulated genes associated with angiogenesis. The heat map is clustered in rows and columns. Scale bar, 100 µm. (S) mRNA expression of ANGPTL4 in HMEC‐1 cells with the control group, CGRP protein‐treated group, CGRP receptor (CLR/RAMP1) knockout group, CGRP receptor knockout + CGRP protein‐treated group. (T) Western blotting of Angptl4 and tubulin in HMEC‐1 cells with WT mice and CGRP−/− knockout mice. (U) ELISA analysis of ANGPTL4 secretion levels in the supernatant culture media of HMEC‐1 treated with CGRP or PBS for 24 h. (V and W) Representative images and quantification of Angptl4 (green)‐ and CD31 (red)‐stained femora from WT and CGRP−/− mice. Scale bar, 100 µm. The data are shown as the mean ± standard deviation. ^*^ *p* < 0.05, ^**^ *p* < 0.01; and ^****^ *p* < 0.0001 by Student's *t*‐test.

H‐type blood vessels are located in the bone marrow near the growth plate; specific high expression of CD31 and EMCN is the hallmark of this blood vessel type. As an important part of the bone microenvironment, H‐type blood vessels have the potential to promote the growth of other surrounding cells, including bone progenitor cells, osteoblasts, chondrocytes, and hematopoietic stem cells [2, 26, 27]. Our previous studies have shown that CGRP can promote angiogenesis and bone formation by activating the FAK–AKT–VEGF signaling pathway, but the relationship between the effects of CGRP on angiogenesis and bone formation remains unclear. We co‐immunostained for CD31 and leptin receptor (LEPR) and found that, compared with those in the control group, the Lepr‐positive BMSCs in the CGRP−/−mice were highly surrounded by bone endothelial cells, and the numbers of CD31‐positive vascular cells and Lepr‐positive BMSCs were lower, indicating that the osteogenic differentiation of bone endothelial cells and BMSCs may be related (Figure 7A,B). To identify the potential factors regulated by CGRP in ECs, we performed a re‐clustering analysis of ECs using scRNA‐seq data (GSE128423). ECs are divided into three clusters: cluster 0, cluster 1, and cluster 2 (Figure 7C). Among these, cluster 0 was enriched in the CGRP high‐expression group (Figure 7D). Ro/e analysis also confirmed that cluster 0 was closely associated with high CGRP expression (Figure 7E). Interestingly, we found that cluster 0 highly expresses ANGPTL4, a key factor regulating angiogenesis and bone resorption [28] (Figure 7F–H), suggesting that ANGPTL4 may serve as a marker molecule for ECs angiogenesis. The characteristic genes of cluster 0 are also primarily enriched in bone differentiation and cell‐cell interactions, such as leukocyte adhesion to vascular endothelial cells and osteoclast differentiation (Figure 7I). Subsequently, the relationship between CGRP and ANGPTL4 was further explored in bulk transcriptomic datasets. Figure 7J–N showed that ANGPTL4 expression increased in parallel with rising CGRP expression levels.

To further validate our findings, we detected differences in mRNA expression levels between the CGRP‐treated group and the control group by RNA‐seq. A total of 2512 differentially expressed genes (log2 fold‐change ≥2) were identified, including 1401 up‐regulated genes and 1111 down‐regulated genes (Figure 7O,P). The common up‐regulated and down‐regulated genes were enriched using the clusterProfiler packages. The top 10 GO terms involved the biological process of angiogenesis and bone differentiation (Figure 7Q). Heatmap analysis of the differentially expressed genes involved in the regulation of angiogenesis showed that ANPEP, MMP2, PDGFA, TNFAIP2, HOXA3, NDNF, RORA, PAK3, ZEB2, NOTCH2, FN1, APOLD1, HSPG2, SMAD3, MYH9, YBX1, SLC12A6, JAG1, ARNT, YAP1, HIF1A, CYP1B1, ADAM8, vascular endothelial growth factor A (VEGFA), EPHB1, EPHB3, EFNA1, ANGPTL6, NOTCH1, THY1, TAZ, ID1, ANGPTL4, IL18, SMAD6, STAT2, and VEGFB were significantly up‐regulated in the CGRP treatment group compared with the control group (Figure 7R), indicating that these molecules, especially ANGPTL4, may serve as potential molecular targets. Taken together, scRNA‐seq data, bulk transcriptome data, and internal RNA‐seq data identified ANGPTL4 as a key factor in regulating angiogenesis and bone resorption.

We measured the mRNA expression of ANGPTL4 in HMEC‐1 cells with a control group, CGRP protein‐treated group, CGRP receptor (CLR/RAMP1) knockout group, and CGRP receptor knockout + CGRP protein‐treated group (Figure 7S). Moreover, protein levels of ANGPTL4 and tubulin in WT mice and CGRP−/−knockout mice were evaluated by western blotting (Figures 7T and S3F). The results found that ANGPTL4 expression was elevated after CGRP protein treatment, was lower in CGRP−/−knockout mice compared to WT mice, and CGRP binds to CLR/RAMP1 receptor to promote the expression of ANGPTL4 in ECs. Figure S3G showed ANGPTL4 expression in ECs of WT and CGRP−/−mice, which was consistent with the aforementioned results. Then, via ELISA of the supernatants from HMEC‐1 cultures, we found that the secreted ANGPTL4 levels were significantly higher in cultures treated with CGRP than those from the control group (Figure 7U). Immunofluorescence staining and quantitative analysis showed that, compared with the control group, CGRP−/−mice had significantly lower Angptl4 expression and weaker CD31 staining (Figure 7V,W). These findings strongly indicated that ANGPTL4 was a potential vascular secretory factor induced by CGRP to regulate vascular endothelial cells, and that ANGPTL4 was expressed in the bone endothelium, which might link angiogenesis and BMSC differentiation.

### 3.8 ANGPTL4 Restores Bone Formation by Coupling With BMSC Osteogenic Differentiation

**FIGURE 8:**
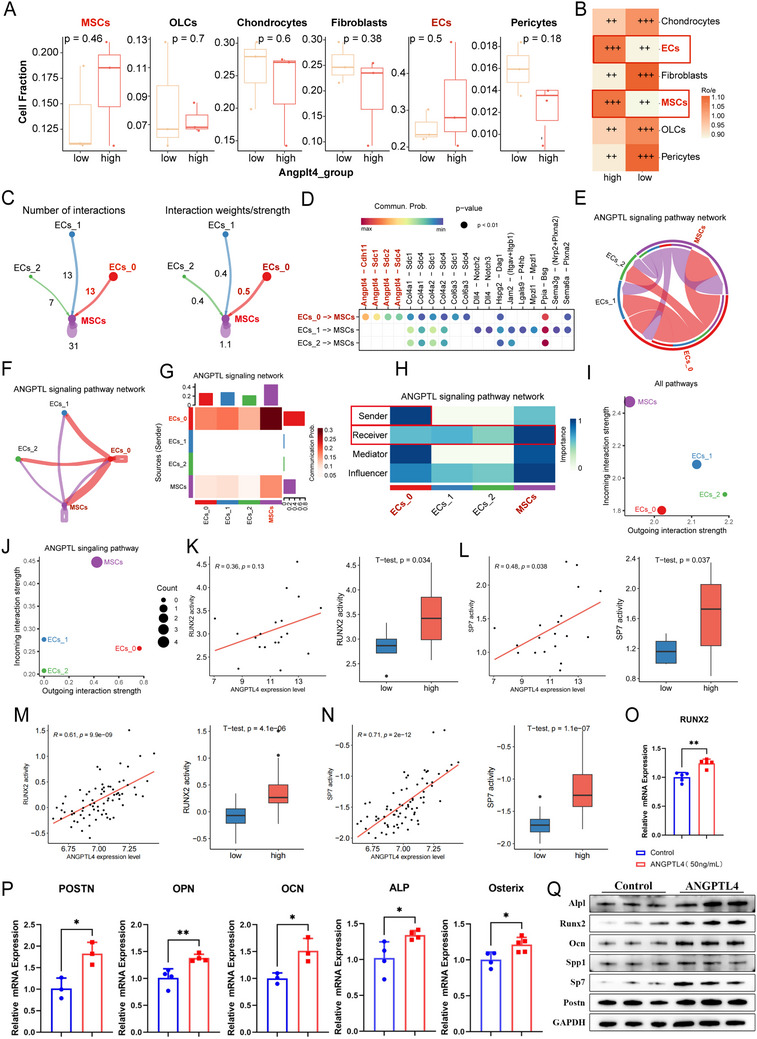
Endothelial cells and bone marrow mesenchymal stem cells maintain close interactions mediated by ANGPTL4. (A) Comparison of MSCs, OLCs, Chondrocytes, Fibroblasts, ECs, and Pericytes between high‐ and low‐CGRP group. (B) Ro/e analysis of various cell types between high‐ and low‐CGRP group. (C) Analysis of cell–cell communication between MSC and EC clusters. (D) Ligand‐receptor interactions in cell–cell communication between MSCs and EC clusters. (E–G) ANGPTL‐related signaling pathway network between MSCs and EC clusters. (H) Signaling role distribution heatmap of the ANGPTL‐related signaling pathway in cell‐cell communication analysis. (I and J) Bubble plot of (I) all pathways (J) ANGPTL signaling pathway interaction profiles across four cell populations (MSCs and EC clusters). Outgoing Interaction Strength: quantifying the cumulative signaling potential of each cell type as a sender. Incoming Interaction Strength: measuring the total signaling receptivity as a receiver. (K) Correlation analysis between RUNX2 transcriptional activity and ANGPTL4 expression levels, and comparison of RUNX2 transcriptional activity between high‐ and low‐BMD groups in GSE35959. (L) Correlation analysis between SP7 transcriptional activity and ANGPTL4 expression levels, and comparison of SP7 transcriptional activity between high‐ and low‐BMD groups in GSE35959. (M) Correlation analysis between RUNX2 transcriptional activity and ANGPTL4 expression levels, and comparison of RUNX2 transcriptional activity between high‐ and low‐BMD groups in GSE56814. (N) Correlation analysis between SP7 transcriptional activity and ANGPTL4 expression levels, and comparison of SP7 transcriptional activity between high‐ and low‐BMD groups in GSE56814. (O and P) RT‐qPCR analysis of Runx2, Postn, Osterix, Opn, Ocn, and Alpl expression levels in bone marrow mesenchymal stem cells (BMSCs) treated with different concentrations of recombinant Angptl4 protein. ^*^ *p* < 0.05, ^**^ *p* < 0.01; and ^***^ *p* < 0.001 by Student's *t*‐test. (Q) Western blotting to determine Alpl, Runx2, Ocn, Spp1, Sp7, Postn, and GAPDH expression levels in BMSCs treated with 50 ng/mL recombinant Angptl4 protein. *n* = 3 mice in each group.

To investigate whether Angptl4 from ECs participates in the regulation of diverse cells within the bone marrow microenvironment. The study first explored the correlation between high and low Angptl4 groups and various cell types. In scRNA‐seq data, ECs and MSCs were enriched in the high Angptl4 group (Figure 8A,B), indicating that Angptl4 is closely associated with both ECs and MSCs. More significantly, cell‐cell interaction analysis revealed that ECs (cluster 0, Angptl4 high expression) exhibited the highest proportion of interactions with MSCs (Number of interactions = 13; Interaction weights/strength = 0.5, Figure 8C), and Angptl4‐related signaling was found to be specifically enriched in EC_0‐MSC interactions (Figure 8D). Within the entire ANGPTL‐related signaling pathway network, EC0‐MSC exhibits the strongest interactions (Figure 8E–G). Among Angptl4‐related signals, EC_0 is the strongest output signal, while MSC is the strongest receiving signal (Figure 8H–J). Interestingly, among all pathways, EC_2 was the strongest signal sender, yet MSCs consistently remains the strongest signal receiver (Figure 8I), further highlighting the potent interaction between the ECs and MSCs. In summary, ECs can regulate BMSCs by secreting Angptl4.

Next, we exploited whether Angptl4 derived from bone ECs regulates the osteogenic and adipogenic differentiation of BMSCs (Figures S4–S9). On one hand, we explored the relationship between Angptl4 expression levels and osteogenic differentiation‐related genes (Alpl, Runx2, Sp1 (known as Opn), Sp7 (known as Osterix), and Postn) expression levels. Bulk transcriptomic data indicate that osteogenic differentiation‐related genes exhibit increased expression alongside rising Angptl4 expression levels. Furthermore, these genes demonstrate higher expression in high‐BMD individuals, middle‐aged patients (compared to elderly patients), and non‐osteoporotic elderly patients (compared to osteoporotic elderly patients) (Figures S4A, S5A, S6A, S7A, and S8A). On the other hand, the relationship between Angptl4 expression levels and adipose differentiation‐related genes (Fabp4, Pparg, Lpl, Plin1 (known as perilipin‐1), Adipoq, and Id4) expression levels was also explored. As Angptl4 expression increased, the expression levels of adipose differentiation‐related genes expression decreased, and they exhibited higher expression in low BMD, elderly patients, and elderly patients with osteoporosis (Figure S4B, S5B, S6B, S7B, and S8B). Next, we further inferred the activity states of transcription factors regulating these genes by bulk transcriptomic gene expression data. And RUNX2, SP7, PPARG, and ID4 exist within the transcription factor‐target gene regulatory network. Similarly, the transcriptional activity of RUNX2 and SP7 increased with rising Angptl4 expression levels (Figures 8I–L and S9A–F). Interestingly, a positive trend emerged between Angptl4 expression and the transcriptional activity of PPARG and ID4 (Figure S9G–P), indicating that Angptl4 regulates their expression levels without affecting their activity. Collectively, these findings indicate that Angptl4 positively regulates the expression and transcriptional activity of osteogenic differentiation‐related genes and negatively regulates the expression of adipose differentiation‐related genes.

**FIGURE 9:**
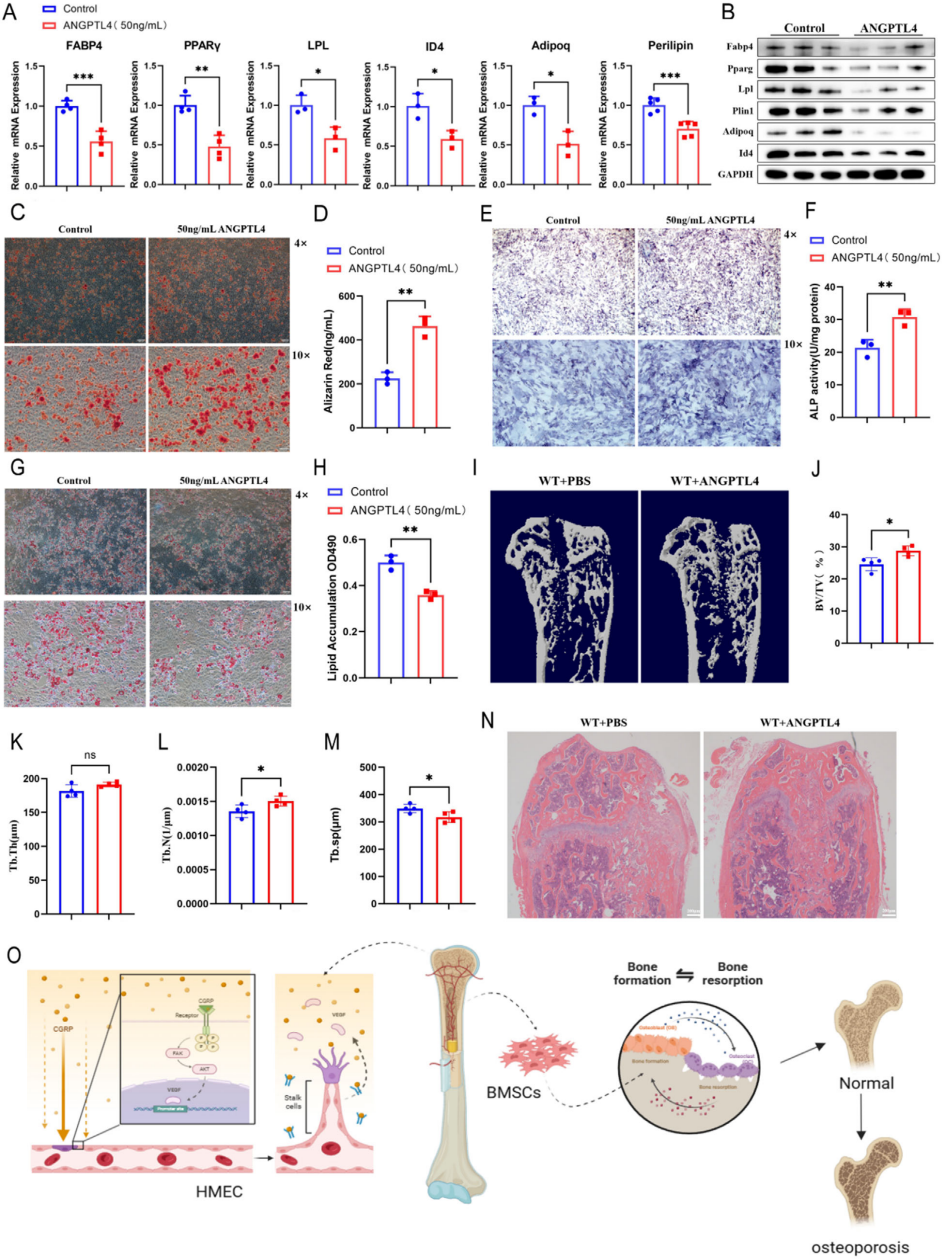
ANGPTL4 secreted by bone vessels restores bone formation by coupling with osteogenic differentiation of bone marrow mesenchymal stem cells. (A) RT‐qPCR analysis of Fabp4, Pparg, LPL, Id4, Adipoq, and perilipin expression levels in bone marrow mesenchymal stem cells (BMSCs) treated with different concentrations of recombinant Angptl4 protein. (B) Western blotting to determine Fabp4, Pparg, Lpl, Plin, Adipoq, Id4, and GAPDH expression levels in BMSCs treated with 50 ng/mL recombinant Angptl4 protein. *n* = 3 mice in each group. (C and D) Representative images of alizarin red S staining and quantification of calcification of BMSCs treated with recombinant Angptl4 protein. (E and F) Representative images of Alpl staining and quantification of Alpl activity in BMSCs treated with recombinant Angptl4 protein. (G and H) Representative images of oil red O staining and quantification of lipid formation by BMSCs treated with recombinant Angptl4 protein. (I–M) Representative micro‐CT images and quantitative micro‐CT analysis of the trabecular bone microarchitecture of WT mice injected with recombinant Angptl4 protein after bone defect introduction. (N) Representative images of hematoxylin–eosin staining in the bone regeneration area of the PBS and Angptl4 treatment groups. (O) Schematic diagram of the effects of CGRP. Scale bar, 1 mm. The data are shown as the mean ± standard deviation. ^*^ *p* < 0.05, ^**^ *p* < 0.01; and ^***^ *p* < 0.001 by Student's *t*‐test. BV/TV, trabecular bone volume per tissue volume; Tb. N, trabecular number; Tb. Sp, trabecular separation; Tb. Th, trabecular thickness.

To further determine the role of Angptl4 in regulating the differentiation of BMSCs, we treated mouse BMSCs with recombinant Angptl4 protein (50 ng/mL) and induced differentiation into osteoblasts and adipocytes in vitro. Quantitative reverse transcription PCR (RT‐qPCR) analysis showed that BMSCs treated with Angptl4 up‐regulated the mRNA expression of osteogenic differentiation‐related genes Alpl, Runx2, Ocn, Spp1, Sp7, and Postn (Figure 8O,P). Western blot analysis further demonstrated that the expression of osteogenic differentiation‐related proteins in the Angptl4‐treated group was higher than that in the control group (Figures 8Q and S10A). And BMSCs treated with Angptl4 down‐regulated the mRNA expression and protein level of adipose differentiation‐related genes Fabp4, Pparg, Lpl, Plin1 (known as perilipin‐1), Adipoq, and Id4 (Figures 9A,B and S10B). The quantitative results of alizarin red staining, Alpl staining, and oil red O staining showed that Angptl4 could promote BMSC formation of more mineralized calcium nodules and secretion of more Alpl (Figure 9C–F). On the contrary, Angptl4 inhibited the formation of fat droplets by BMSCs (Figure 9G,H). Therefore, Angptl4 promoted the differentiation of primary BMSCs into osteoblasts and inhibited their differentiation into adipocytes in vitro. These data suggested that CGRP could promote the secretion of ANGPTL4 from bone vessels and the differentiation of BMSCs into osteoblasts, and inhibit BMSC differentiation into adipocytes.

Next, we evaluated if Angptl4 promoted trabecular bone formation in in vivo mice with bone defects. WT mice (3 months old) with bone defects were injected with recombinant Angptl4 protein (6 mg/kg) via the tail vein every 2 days, and the control group was injected with the same volume of PBS solution via the same route. The femora of the mice were removed on day 7 for subsequent analysis. Micro‐CT analysis showed that recombinant Angptl4 protein promoted bone regeneration in the bone defect area and promoted trabecular formation (Figure 9I–M). Hematoxylin–eosin staining demonstrated that recombinant Angptl4 protein promoted the formation of trabecular bone (Figure 9N). Additionally, this treatment led to fewer TRAP‐positive osteoclasts. These data suggested that the bone‐derived angiogenic factor ANGPTL4 actively participated in bone repair within the bone regeneration area, facilitating bone formation. Figure 9O illustrates the schematic diagram of the effects of CGRP in this study.

## 4 Discussion

The vascular–osteogenic coupling in the skeletal system relies on coordinated spatial and temporal interactions. During the process of bone defect repair, the newly formed bone vasculature provides nourishment for osteogenesis and transports osteogenic‐related factors to the bone regeneration area, thereby promoting bone healing [29]. Research has shown that CGRP not only stimulates osteoblast differentiation but also acts as a bone metabolism regulator by inhibiting osteoclastogenesis mediated through the OPG/RANKL pathway [30]. Appelt et al.,[31] demonstrated that CGRP‐deficient mice exhibit profoundly impaired bone regeneration. They also observed that CGRP and the CGRP receptor are expressed in regenerating bone at notable levels, especially during the early and intermediate stages of bone regeneration [31]. Our group has previously found that CGRP is a key regulator of age‐dependent BMSC transition to osteoblast or adipocyte differentiation, and recombinant CGRP can partially restore bone formation in aged mice and OVX mice [7]. Despite an increasing number of studies confirming the effects of CGRP on bone formation and angiogenesis, the underlying complex mechanisms remain largely unexplored. This study found that CGRP promoted angiogenesis through the FAK–AKT–VEGF signaling pathway and increased secretion of ANGPTL4 in bone vessels, which may comprise the main mechanism of CGRP‐induced bone formation.

Our previous studies have shown that plasma CGRP levels in patients with femoral neck fractures markedly rise upon admission and continue to increase after 24 h, suggesting that stimulating CGRP secretion may be the body's healing mechanism in response to mechanical injury [7]. Disappointingly, the expression levels of CGRP in humans at different ages have not been explored. In the present study, we observed that CGRP levels in humans exhibit a significant downward trend with decreasing bone mass, and also tend to decrease with increasing age. This finding underscores the strong correlation between CGRP levels in the body and age, as well as bone mass, further highlighting the positive role of CGRP in osteoporosis and bone regeneration. However, large‐scale clinical studies are required to determine whether CGRP can serve as a biomarker for osteoporosis and offer clinical guidance.

This study utilized bulk transcriptome data, scRNA‐seq data, and phosphoproteome data to reveal that CGRP promotes the migration and tube formation of HMEC‐1, and discovered that the FAK–AKT–VEGF signaling pathway is the primary mechanism underlying CGRP‐mediated angiogenesis. It was demonstrated that 10^−9 ^mol/L CGRP could promote angiogenesis in human umbilical vein endothelial cells [32]. Consistent with their findings, we determined that 50 ng/mL CGRP was the optimal concentration to promote angiogenesis and chose this concentration for subsequent experiments. Several studies have shown that CGRP directly stimulates angiogenesis in vivo by activating the CGRP receptor [33, 34]. Moreover, CGRP could also promote angiogenesis by activating the AMPK–eNOS pathway in endothelial cells [35]. These findings are similar to our results, and our study comprehensively revealed the mechanism by which CGRP promotes angiogenesis through the FAK–AKT–VEGF signaling pathway, identifying a novel signaling pathway for CGRP's pro‐angiogenic role.

Next, in vivo experiments further confirmed the beneficial effects of CGRP in promoting bone angiogenesis and its coupling with the osteogenic process. We found that, after knocking out the CGRP gene, the number of CD31^high^Emcn^high^ endothelial cells and the expression of VEGFA, a marker of angiogenesis, were lower than in the bone marrow of WT mice, resulting in reduced angiogenesis. Additionally, the absence of CGRP led to decreased bone mass in these mice. We also constructed a bone defect model and observed that bone neovascularization was slower and bone regeneration capacity was poorer in CGRP−/− mice. Niedermair et al. [36] reported that mice lacking αCGRP exhibited reduced bone mass. Appelt et al. [31] revealed that αCGRP−/− mice had reduced bone regeneration capacity after fracture. Using a segmental bone defect model, Ye et al. [33] found that CGRP could concentration‐dependently enhance endothelial cell migration and tube formation, promote the phosphorylation of FAK at the Y397 site, and elevate the expression of VEGFA, which has significantly contributed to the clinical translation of osteoporosis treatment. However, these studies did not comprehensively explore the underlying molecular mechanisms. Moreover, unlike these studies, we used mice with a complete knockout of the CGRP gene and obtained similar results. Our study further demonstrated the impact of CGRP deficiency on bone angiogenesis and the pro‐bone repair activity of CGRP after injury via enhancement of osteoblast proliferation, thereby providing deeper insights into the mechanism of CGRP in the bone formation process, specifically through the angiogenesis–osteogenesis coupling mechanism.

To further explore the underlying mechanisms of CGRP in bone formation, we investigated the effects of exogenous CGRP on bone regeneration in mice with bone defects. As expected, CGRP promoted angiogenesis in the bone injury area, increased bone mass and trabecular bone volume, and facilitated bone regeneration after injury. These positive effects may be mediated by several mechanisms: (1) enhanced osteoblast function, characterized by increased trabecular bone and elevated expression levels of Runx2 associated with osteoblasts; (2) the decrease in the number of osteoclasts. The study by Zhang and colleagues [37] found that knockdown in vivo of the CGRP‐receptor‐encoding genes Calcrl or Ramp1 substantially reversed the magnesium‐induced osteogenesis in rats with ovariectomy‐induced osteoporosis. Interestingly, overexpression of these genes could also promote magnesium‐induced osteogenesis. Luo et al. [38] showed that porous microspheres loaded with CGRP effectively alleviated inflammation‐induced BMSC dysfunction and promoted alveolar bone regeneration by releasing CGRP. More importantly, our study not only further confirmed the crucial role of CGRP in bone formation but also provided the first in vivo evidence in a bone defect model of the promotional effect of exogenous CGRP on bone formation. Therefore, CGRP treatment may serve as a promising strategy for supporting bone angiogenesis and improving fracture healing.

In this study, scRNA‐seq data and internal RNA‐seq identify the key mediator, namely ANGPTL4, connecting angiogenesis and bone formation. Significant differences in ANGPTL4 expression were further confirmed in CGRP‐deficient and WT animals. In vitro experiments confirmed that ANGPTL4 mRNA expression levels were significantly increased upon CGRP treatment, and CGRP promoted ANGPTL4 protein secretion from HMEC‐1, which was consistent with the results of scRNA‐seq and RNA‐seq. It was speculated that another possible mechanism by which CGRP promotes bone formation is the stimulation of vascular endothelial cells to secrete ANGPTL4, a secretory angiogenin‐like protein that affects angiogenesis and lipid metabolism [39, 40, 41]. The PI3K/Akt and FAK/ERK1/2 signaling were demonstrated as a nexus with ANGPTL4 secretion [42], further demonstrating our conclusion that CGRP promotes ECs to secrete ANGPTL4 through FAK–AKT–VEGF signaling pathways. In addition, ANGPTL4 has been detected in osteoblasts and osteoclasts, in which it affects the bone resorption process and osteoclast formation [43, 44]. In the process of alveolar bone remodeling, ANGPTL4 positively correlates with the osteogenic differentiation of periodontal ligament stem cells [45]. After tenotomy, ANGPTL4 can be induced from brown adipose tissue to promote chondrogenesis [46]. Various studies have shown that ANGPTL4 may participate in the process of bone formation and act as a mediator of the effect of CGRP on bone remodeling. The role of ANGPTL4 in bone formation was verified in animal experiments. CGRP−/− mice showed significantly reduced Angptl4 expression and weaker CD31 staining, strongly suggesting that ANGPTL4 could be a potential angiogenic factor secreted by CGRP‐stimulated vascular endothelial cells. ANGPTL4 is expressed in bone endothelium and may couple angiogenesis with BMSC differentiation. Taking these results together, it can be hypothesized that another possible mechanism by which CGRP promotes bone formation is the stimulation of the secretion of ANGPTL4. This strongly suggests that ANGPTL4 is a potential angiogenic factor secreted by vascular endothelial cells in response to CGRP, and that it is expressed in bone endothelium, which may couple angiogenesis and BMSC differentiation.

Then, we further examined the role of ANGPTL4 in osteogenesis. ScRNA‐seq data reveal potent cellular interactions between ECs and MSCs, and that ECs can regulate BMSCs by secreting Angptl4. In scRNA‐seq data and cellular experiments, Angptl4 promoted the differentiation of BMSCs into osteoblasts and up‐regulated the expression of osteogenic differentiation‐related genes and proteins, such as Opn, Alpl, and Runx2. Alizarin red and Alpl staining showed that ANGPTL4 promoted osteogenic differentiation. Conversely, Angptl4 inhibited the adipogenic differentiation of BMSCs and down‐regulated the expression of adipogenic differentiation‐related genes such as Fabp4 and Pparγ. Oil red O staining indicated a reduction in lipid droplets, revealing for the first time the role and mechanism of ANGPTL4 in bone formation. At the same time, we found that CGRP deficiency downregulated the expression of Angptl4 in the bone marrow and reduced CD31 expression, further supporting the relationship between ANGPTL4, CGRP, and bone blood vessels. Subsequently, in vivo experiments using a bone defect model with exogenous administration of recombinant Angptl4 protein showed increased expression of Ocn‐positive osteoblasts and Runx2 in the Angptl4 group, whereas the number of osteoclasts decreased and bone resorption was weakened in these mice. This study revealed the role of ANGPTL4 in promoting bone formation in the bone regeneration area and confirmed that the bridge between CGRP promoting bone angiogenesis and bone formation may be the secreted protein ANGPTL4. It is worth noting that Ang2 promotes angiogenesis in tissue‐engineered bone and improves the repair of bone defects by inducing autophagy. Homology comparison showed that ANGPTL4 and human ANG2 have 42% homology at the amino acid level, and their similar structures might be the reason for their similar activity [47]. Nevertheless, this study also has certain limitations, namely the need for further investigation of the long‐term efficacy of CGRP therapy in larger animal models (segmental defects of long bones) or clinical trials. And the bone regeneration assays in this study is limited to short‐term assessments. Future research should incorporate the mechanical strength of regenerated bone and extend regeneration analysis to longer times. Moreover, Liu et al. [48] constructed the trifunctional peptide coating (MPN@K6‐RGD/OGP/Ang), demonstrating positive effects on bone tissue repair and holding significant potential for clinical applications in orthopedic and dental implants. This further suggests that subsequent research could explore the role of nanomaterials‐based ANGPTL4.

## 5 Conclusions

This comprehensive study revealed that CGRP, a neuropeptide, can promote the formation of H‐type blood vessels and induce bone vascular endothelial cells to secrete ANGPTL4 through the activation of the FAK–AKT–VEGF signaling pathway. ANGPTL4 secreted by bone vascular endothelial cells positively regulates the osteogenic differentiation of BMSCs and negatively regulates their adipogenic differentiation, thereby facilitating the process of bone regeneration. The novel mechanism we have proposed provides novel evidence for the pro‐osteogenic effects of CGRP. The results of this study offer a compelling treatment strategy for supporting fracture healing and repair of bone defects caused by surgeries, trauma, or osteoporosis.

## Funding

This work was supported by the Natural Science Foundation of Hunan Province (2024JJ5494), Natural Science Foundation of Changsha City (Kq2403094), Hunan Provincia Development and Reform Commission of Innovative Research Program (2021‐212‐23), Hunan Innovative Province Construction Special Project (2021ZK4025), and the Hunan Provincial Department of Finance Gra (2024‐31, 45, 2022‐151, 2021‐139 and 2020‐83).

## Ethics Statement

This study was approved by the Ethics Committee of the Second Xiangya Hospital of Central South University (ethics approval number Kq2208340).

## Consent

Written informed consent for publication was obtained from all participants.

## Conflicts of Interest

The authors declare no conflicts of interest.

## Supporting information




**Supporting File**: advs73538‐sup‐0001‐SuppMat.docx.

## Data Availability

The data that support the findings of this study are available in the supplementary material of this article.
