# Supplementary material for: CGRP Enhances the Regeneration of Bone Defects by Regulating Bone Marrow Mesenchymal Stem Cells Through Promoting ANGPTL4 Secretion by Bone Blood Vessels
Source: Adv Sci (Weinh). 2026 Jan 5;13(14):e22295. doi: 10.1002/advs.202522295 (PMC12970192; doi:10.1002/advs.202522295)
Supplement: Supplementary file 1 — Supporting File: advs73538‐sup‐0001‐SuppMat.docx. [file ADVS-13-e22295-s001.docx]

**Supplementary figures**


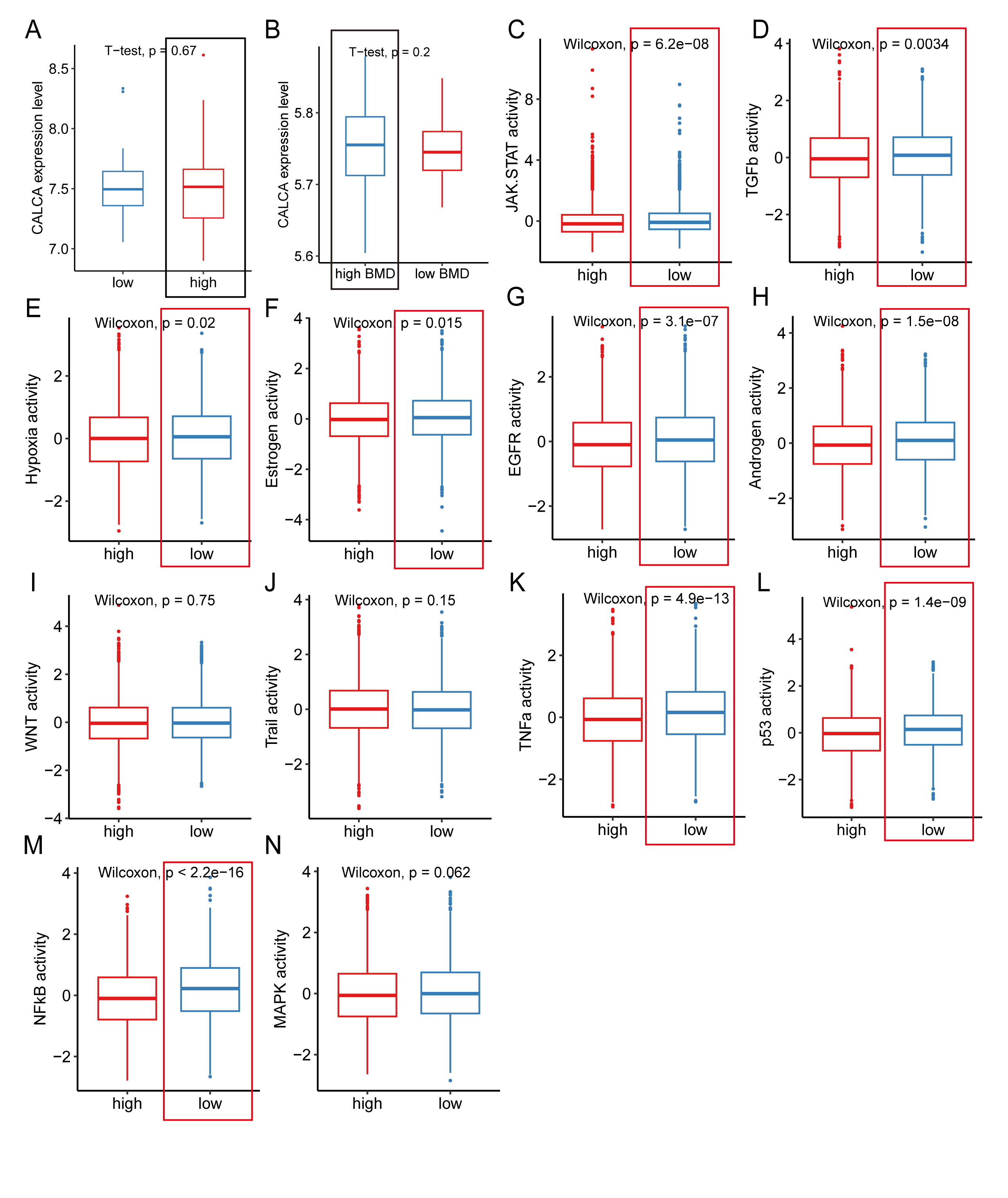


**Figure S1. Expression of CGRP in patients and the activity score for signaling pathways in scRNA-seq data.** **(A-B)** CGRP expression in patients with high and low BMD in **(A)** GSE56814, **(B)** GSE56815. **(C)** JAK-STAT pathway activity, **(D)** TGFβ pathway activity, **(E)** Hypoxia pathway activity, **(F)** Estrogen pathway activity, **(G)** EGFR pathway activity, **(H)** Androgen pathway activity, **(I)** WNT pathway activity, **(J)** TRAIL pathway activity, **(K)** TNFα pathway activity, **(L)** p53 pathway activity, **(M)** NF-kβ pathway activity, and **(N)** MAPK pathway activity in high- and low-CGRP groups in scRNA-seq data.


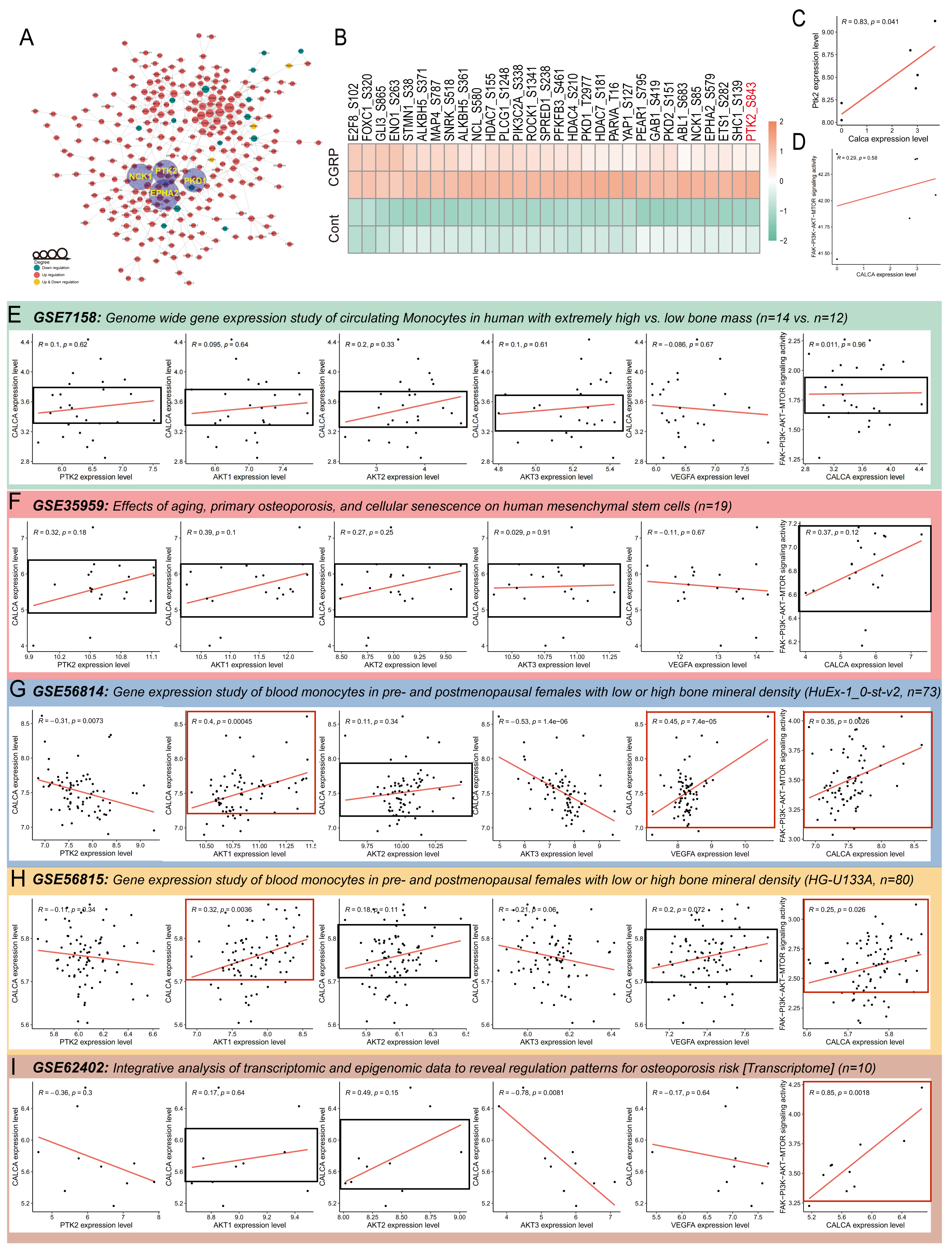


**Figure S2. The correlation between CGRP expression and the FAK–AKT–VEGF signaling pathway. (A)** The GO term analysis of common up-regulated and down-regulated phosphoproteins was performed using the package clusterProfiler. The top 19 GO terms are displayed. **(B)** The heatmap shows the up-regulated phosphoproteins associated with angiogenesis. **(C)** Correlation analysis between CGRP expression and PTK2 expression. **(D)** Correlation analysis between CGRP expression and FAK-PI3K-AKT-MTOR signaling activity. **(E)** Correlation analysis between CGRP expression and PTK2 expression/Akt1 expression/Akt2 expression/Akt3 expression/Vegfa expression /FAK-PI3K-AKT-MTOR signaling activity in GSE7159. **(F)** Correlation analysis between CGRP expression and PTK2 expression/Akt1 expression/Akt2 expression/Akt3 expression/Vegfa expression /FAK-PI3K-AKT-MTOR signaling activity in GSE35959. **(G)** Correlation analysis between CGRP expression and PTK2 expression/Akt1 expression/Akt2 expression/Akt3 expression/Vegfa expression /FAK-PI3K-AKT-MTOR signaling activity in GSE56814. **(H)** Correlation analysis between CGRP expression and PTK2 expression/Akt1 expression/Akt2 expression/Akt3 expression/Vegfa expression /FAK-PI3K-AKT-MTOR signaling activity in GSE56815. **(I)** Correlation analysis between CGRP expression and PTK2 expression/Akt1 expression/Akt2 expression/Akt3 expression/Vegfa expression /FAK-PI3K-AKT-MTOR signaling activity in GSE62402.


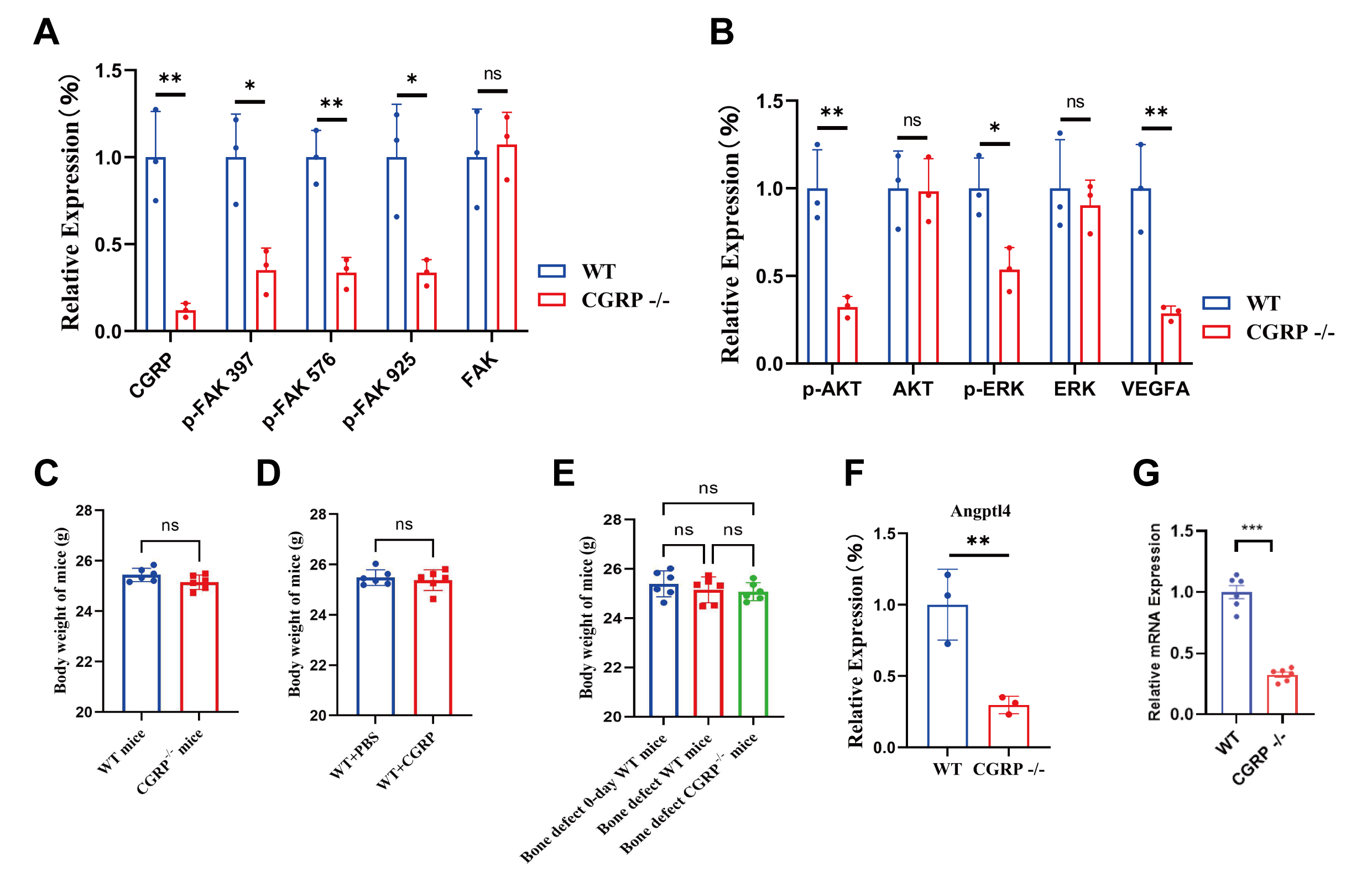


**Figure S3. Western blotting quantification results of WT mice and CGRP−/− knockout mice and weight analysis for mice in different group. (A-B)** Western blotting of CGRP, p-FAK 397, p-FAK 576, p-FAK 925, FAK, p-AKT, AKT, p-ERK, ERK, VEGFA, and tubulin in HMEC-1 cells with WT mice and CGRP−/− knockout mice. **(C-E)** Weight analysis for mice in each group. **(F)** Western blotting of Angptl4 and tubulin in HMEC-1 cells with WT mice and CGRP−/− knockout mice. **(G)** Quantitative reverse transcription polymerase chain reaction (RT-qPCR) analysis of ANGPTL4 expression in HMEC-1 treated with CGRP or PBS for 24 h.


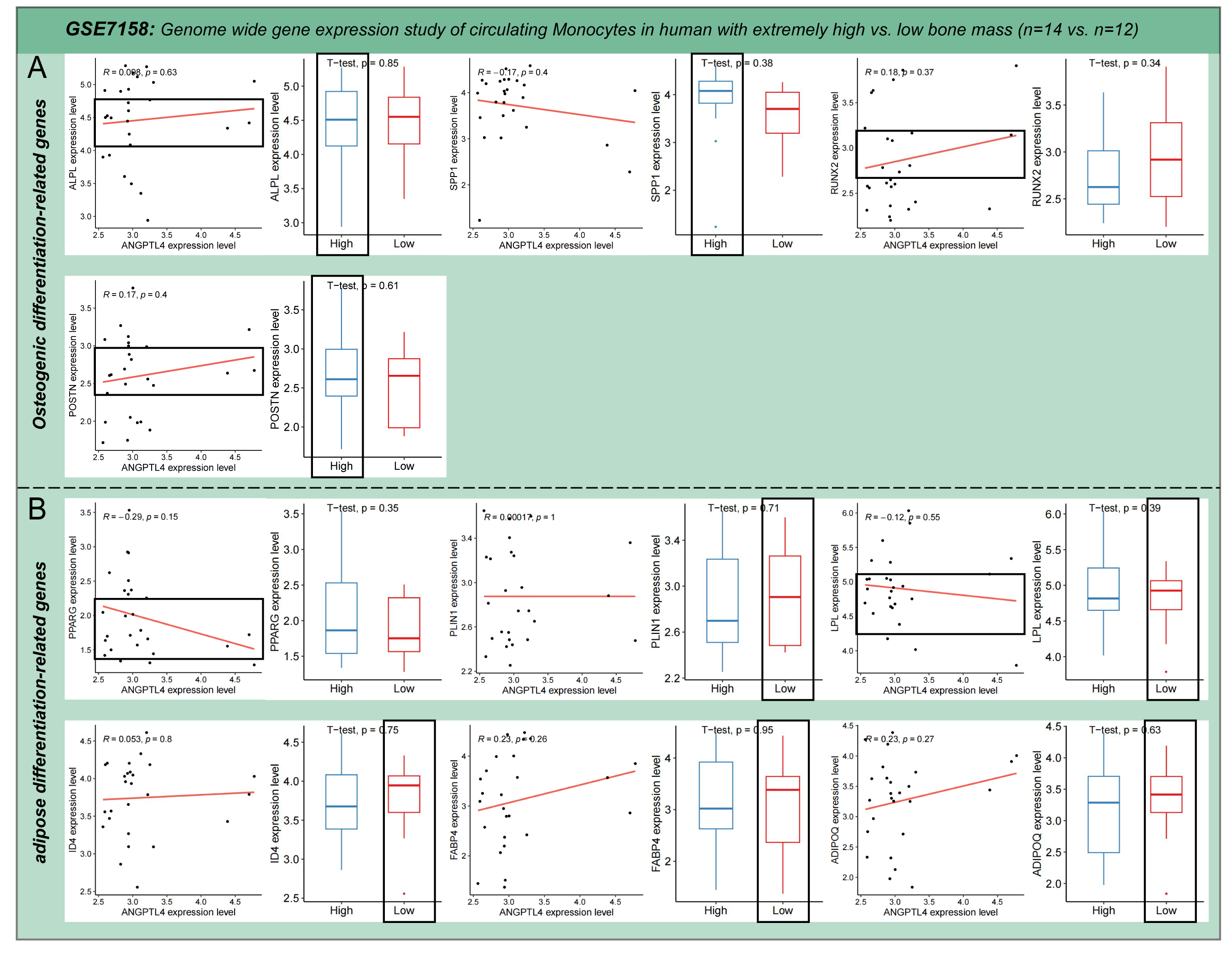


**Figure S4. Angptl4 derived from bone ECs regulates the osteogenic and adipogenic differentiation of BMSCs in GSE7158. (A)** The relationship between Angptl4 expression levels and osteogenic differentiation-related genes (Alpl, Runx2, Sp1 (known as Opn), Sp7 (known as Osterix), and Postn) expression levels. And expression levels of Alpl, Runx2, Sp1, Sp7, and Postn in high- and low-BMD groups. **(B)** The relationship between Angptl4 expression levels and adipose differentiation-related genes (Fabp4, Pparg, Lpl, Plin1 (known as perilipin-1), Adipoq, and Id4) expression levels. And expression levels of Fabp4, Pparg, Lpl, Plin1, Adipoq, and Id4 in high- and low-BMD groups.


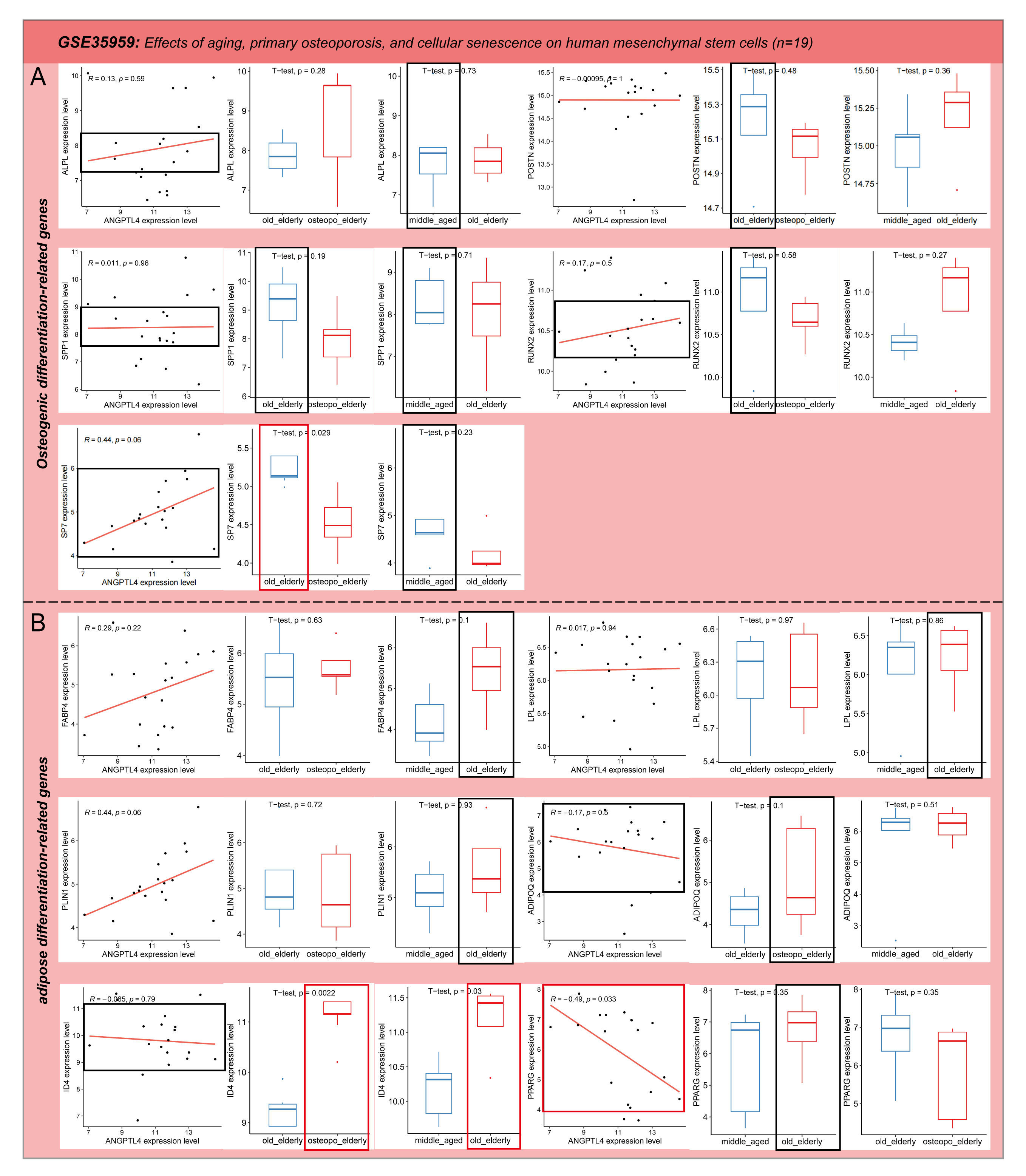


**Figure S5. Angptl4 derived from bone ECs regulates the osteogenic and adipogenic differentiation of BMSCs in GSE35959. (A)** The relationship between Angptl4 expression levels and osteogenic differentiation-related genes (Alpl, Runx2, Sp1 (known as Opn), Sp7 (known as Osterix), and Postn) expression levels. Expression levels of Alpl, Runx2, Sp1, Sp7, and Postn in middle-aged and elderly groups, non-osteoporotic and osteoporotic elderly groups. **(B)** The relationship between Angptl4 expression levels and adipose differentiation-related genes (Fabp4, Pparg, Lpl, Plin1 (known as perilipin-1), Adipoq, and Id4) expression levels. Expression levels of Fabp4, Pparg, Lpl, Plin1, Adipoq, and Id4 in in middle-aged and elderly groups, non-osteoporotic and osteoporotic elderly groups.


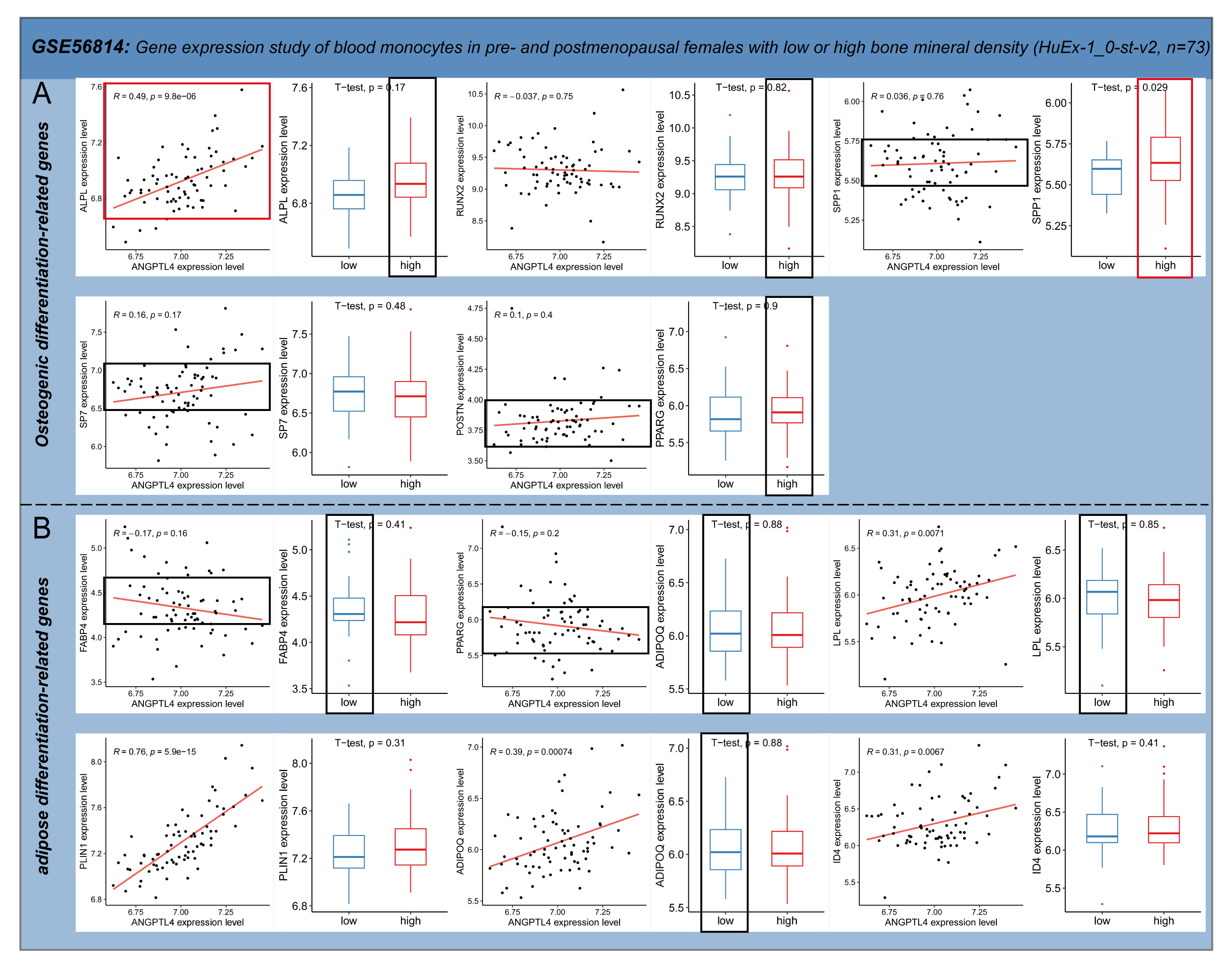


**Figure S6. Angptl4 derived from bone ECs regulates the osteogenic and adipogenic differentiation of BMSCs in GSE56814. (A)** The relationship between Angptl4 expression levels and osteogenic differentiation-related genes (Alpl, Runx2, Sp1 (known as Opn), Sp7 (known as Osterix), and Postn) expression levels. And expression levels of Alpl, Runx2, Sp1, Sp7, and Postn in high- and low-BMD groups. **(B)** The relationship between Angptl4 expression levels and adipose differentiation-related genes (Fabp4, Pparg, Lpl, Plin1 (known as perilipin-1), Adipoq, and Id4) expression levels. And expression levels of Fabp4, Pparg, Lpl, Plin1, Adipoq, and Id4 in high- and low-BMD groups.


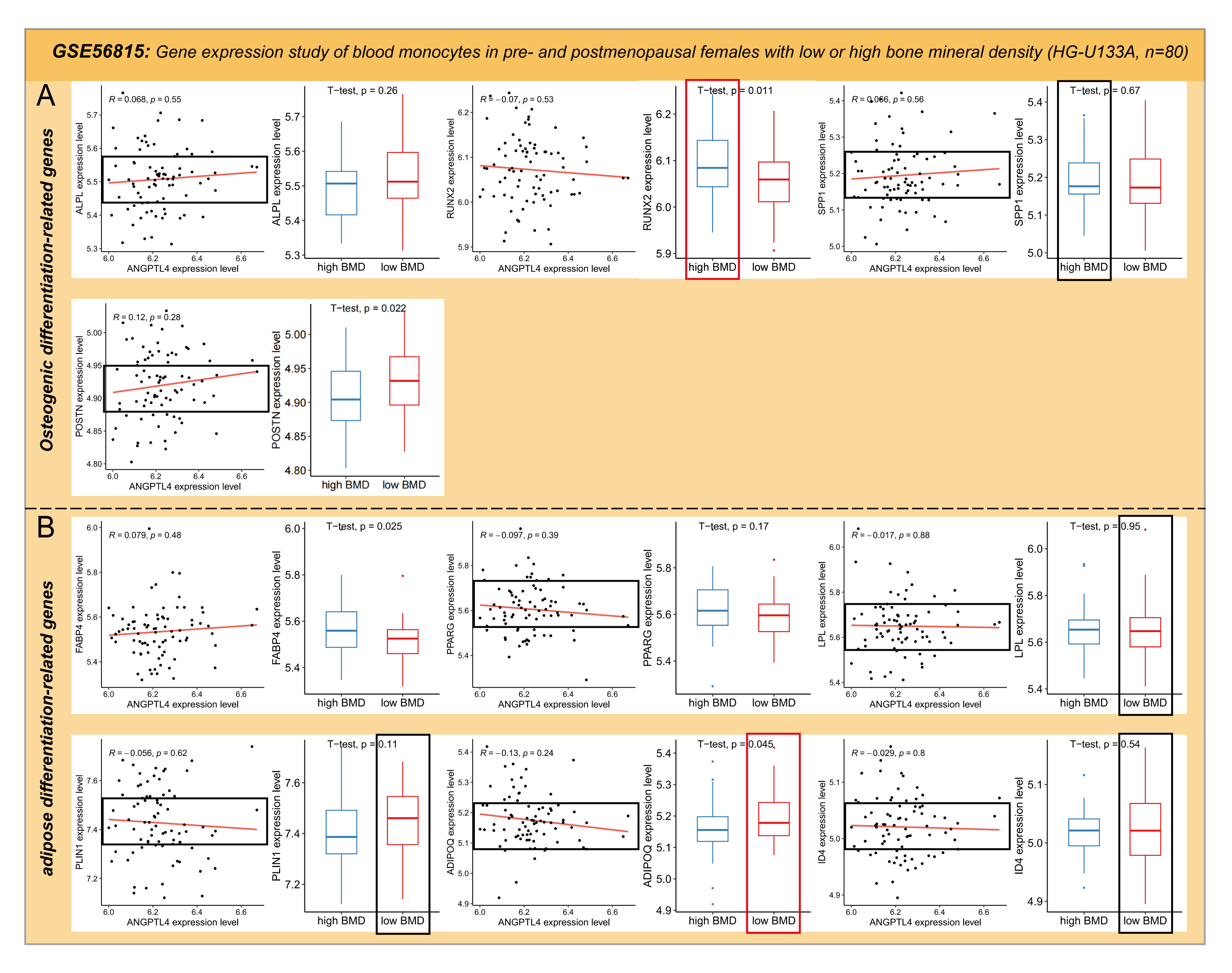


**Figure S7. Angptl4 derived from bone ECs regulates the osteogenic and adipogenic differentiation of BMSCs in GSE56815. (A)** The relationship between Angptl4 expression levels and osteogenic differentiation-related genes (Alpl, Runx2, Sp1 (known as Opn), Sp7 (known as Osterix), and Postn) expression levels. And expression levels of Alpl, Runx2, Sp1, Sp7, and Postn in high- and low-BMD groups. **(B)** The relationship between Angptl4 expression levels and adipose differentiation-related genes (Fabp4, Pparg, Lpl, Plin1 (known as perilipin-1), Adipoq, and Id4) expression levels. And expression levels of Fabp4, Pparg, Lpl, Plin1, Adipoq, and Id4 in high- and low-BMD groups.


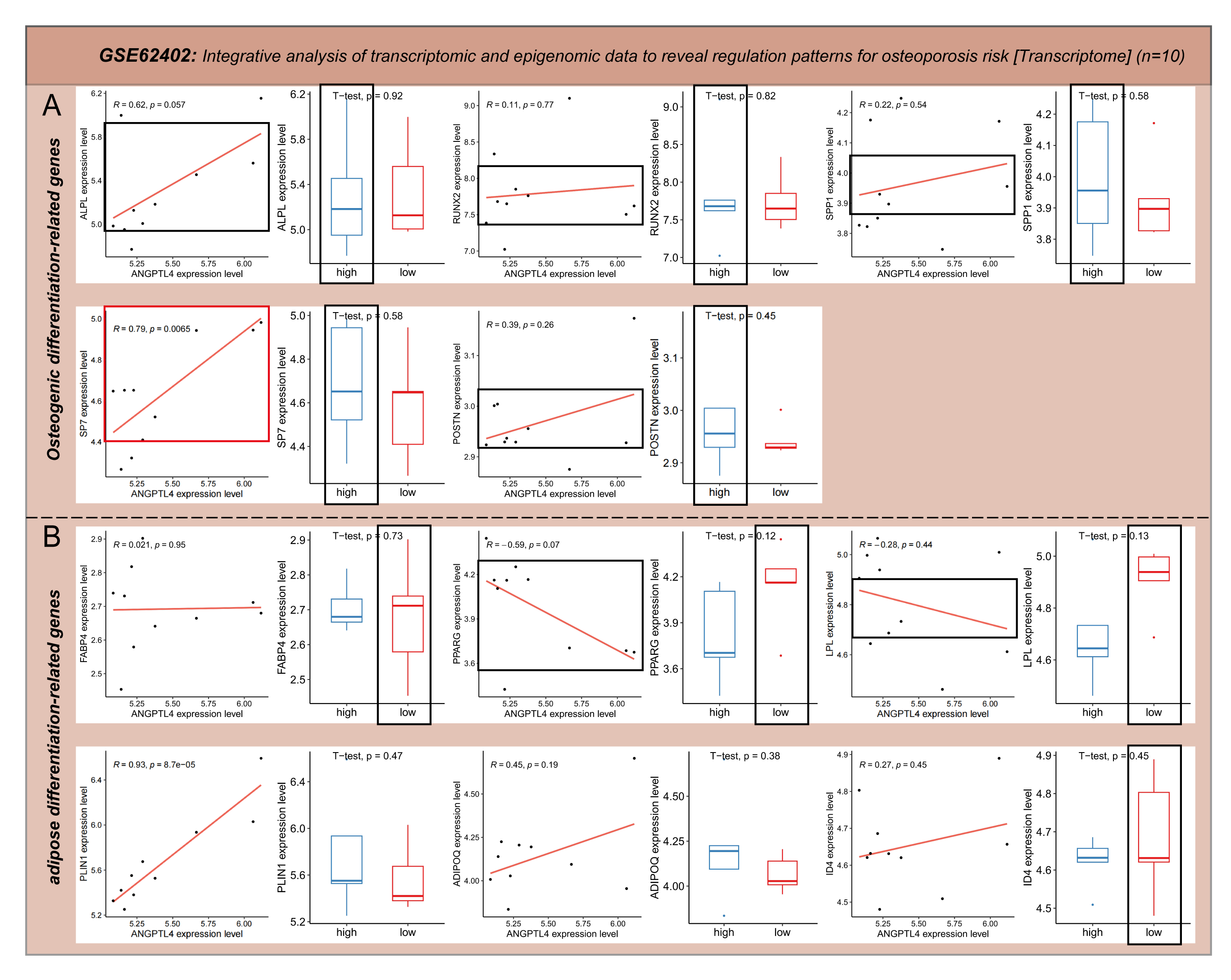


**Figure S8. Angptl4 derived from bone ECs regulates the osteogenic and adipogenic differentiation of BMSCs in GSE62402. (A)** The relationship between Angptl4 expression levels and osteogenic differentiation-related genes (Alpl, Runx2, Sp1 (known as Opn), Sp7 (known as Osterix), and Postn) expression levels. And expression levels of Alpl, Runx2, Sp1, Sp7, and Postn in high- and low-BMD groups. **(B)** The relationship between Angptl4 expression levels and adipose differentiation-related genes (Fabp4, Pparg, Lpl, Plin1 (known as perilipin-1), Adipoq, and Id4) expression levels. And expression levels of Fabp4, Pparg, Lpl, Plin1, Adipoq, and Id4 in high- and low-BMD groups.


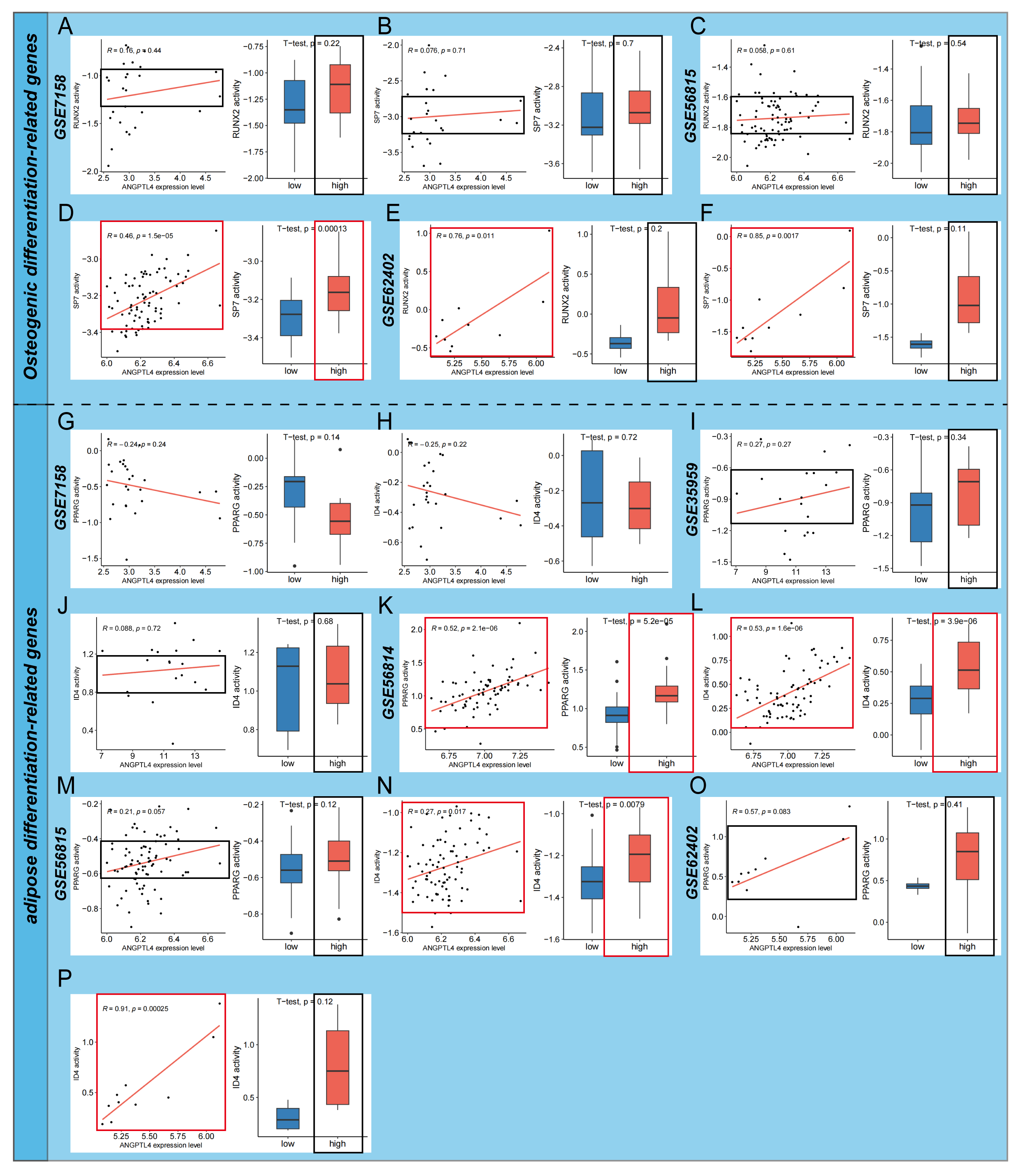


**Figure S9. Angptl4 derived from bone ECs regulates the osteogenic and adipogenic differentiation of BMSCs. (A-F)** The correlation between Angptl4 expression levels and the RUNX2/SP7 transcription factors activity, and RUNX2/SP7 transcription factors activity levels in high- and low-ANGPTL4 groups in **(A-B)** GSE7158, **(C-D)** GSE56815, and **(E-F)** GSE62402. **(G-P)** The correlation between Angptl4 expression levels and the PPARG/ID4 transcription factors activity, and PPARG/ID4 transcription factors activity levels in high- and low-ANGPTL4 groups in **(G-H)** GSE7158, **(I-J)** GSE56815, **(K-L)** GSE56814, **(M-N)** GSE56815, and **(O-P)** GSE62402.

**
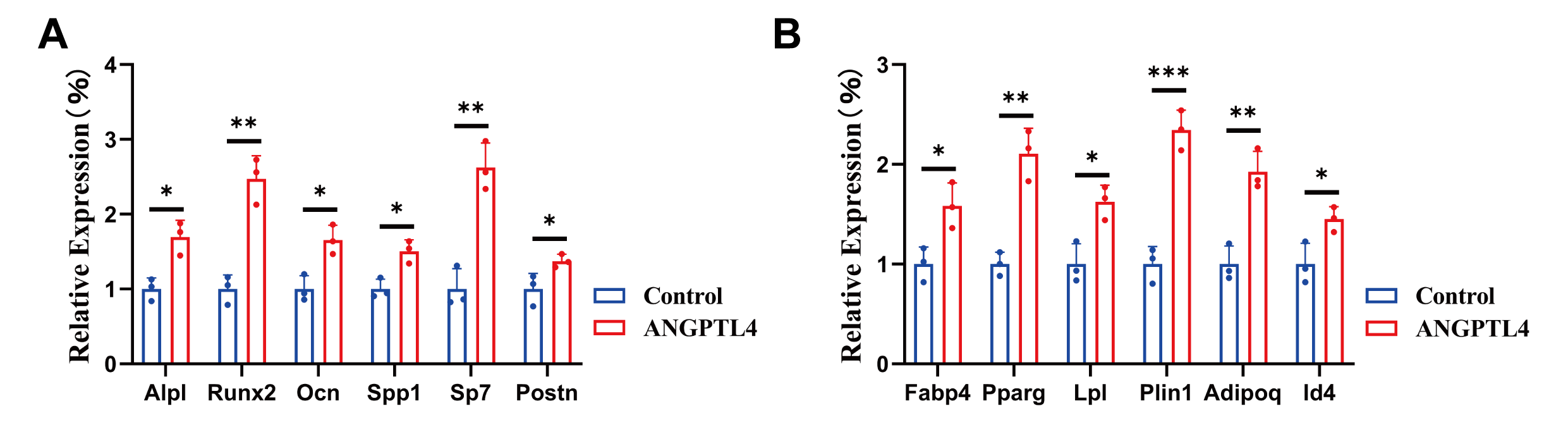
Figure S10. Western blotting quantification results of osteogenic differentiation-related genes and adipose differentiation-related genes. (A)** Western blotting to determine Alpl, Runx2, Ocn, Spp1, Sp7, Postn, and GAPDH expression levels in BMSCs treated with 50 ng/mL recombinant Angptl4 protein. n = 3 mice in each group. **(B)** Western blotting to determine Fabp4, Pparg, Lpl, Plin, Adipoq, Id4, and GAPDH expression levels in BMSCs treated with 50 ng/mL recombinant Angptl4 protein. n = 3 mice in each group.
